# Supplementary material for: Sexual selection in females and the evolution of polyandry
Source: PLoS Biol. 2023 Jan 10;21(1):e3001916. doi: 10.1371/journal.pbio.3001916 (PMC9831318; doi:10.1371/journal.pbio.3001916)
Supplement: S1 Text — Fig A. Imbalance between studies of sexual selection in males and females. Fig B. Methodological predictors of female Bateman gradients. Fig C. Egger’s regression. Fig D. Phylogenetic tree of all sampled species. Fig E. Significance of the used effect size. Table A. Global tests of sexual selection in females using restricted maximum likelihood REML approach. Table B. Comparison of Bateman gradients among major taxonomic groups. Table C. Predictors of interspecific variation in female Bateman gradients using restricted maximum likelihood (REML) approach. Table D. Estimates of polyandry and mating system classification (low-polyandry versus high-polyandry). Table E. Global tests of sexual selection in females excluding sex-role reversed species. Table F. Determinants of sexual selection in females excluding sex-role reversed species. Table G. Global tests of sexual selection in females excluding humans. Table H. Determinants of sexual selection in females excluding humans. (PDF) [file pbio.3001916.s001.pdf]

Supplementary Information – S1 Text

Sexual selection in females and the evolution of polyandry

Salomé Fromonteil[1¶], Lucas Marie-Orleach[2,3¶], Lennart Winkler[4], Tim Janicke[1,4*]

[1] CEFE, Univ Montpellier, CNRS, EPHE, IRD, Montpellier, France.

[2] Natural History Museum, University of Oslo, Oslo, Norway.

[3] CNRS, Université de Rennes 1, ECOBIO (Écosystèmes, biodiversité, évolution) - UMR 6553, Rennes, France.

[4] Applied Zoology, TU Dresden, Dresden, Germany.

¶ Both authors contributed equally to this work.

* tim.janicke@cefe.cnrs.fr

This Supplementary Information file includes: Figures A - E

Tables A - H

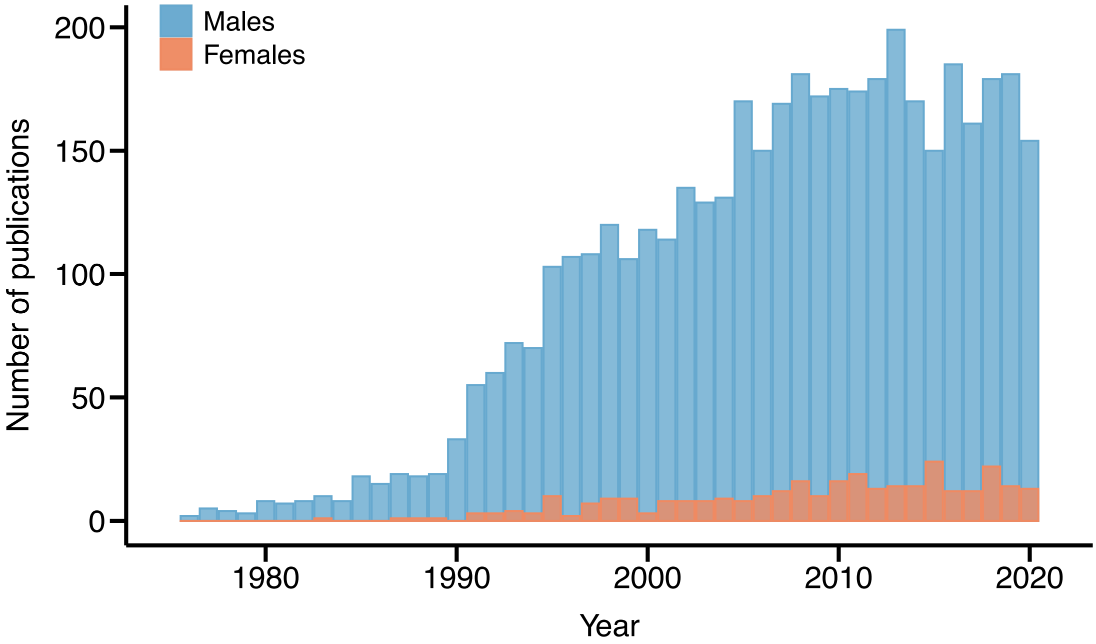

Figure A. Imbalance between studies of sexual selection in males and females. Bars indicate a strong male bias in the number of published articles on sexual selection indexed in ISI Web of Science (Clarivate Analytics) between 1900-2021. Data obtained from topic search using the search terms "sexual selection AND (male choice OR female competition)" for female and "sexual selection AND female choice OR male competition)" for male search. This is not meant to provide an exhaustive search of publications on sexual selection but to showcase the publication bias towards male studies focusing on Darwinian sexual selection in terms of competition for and choice of mating partners.

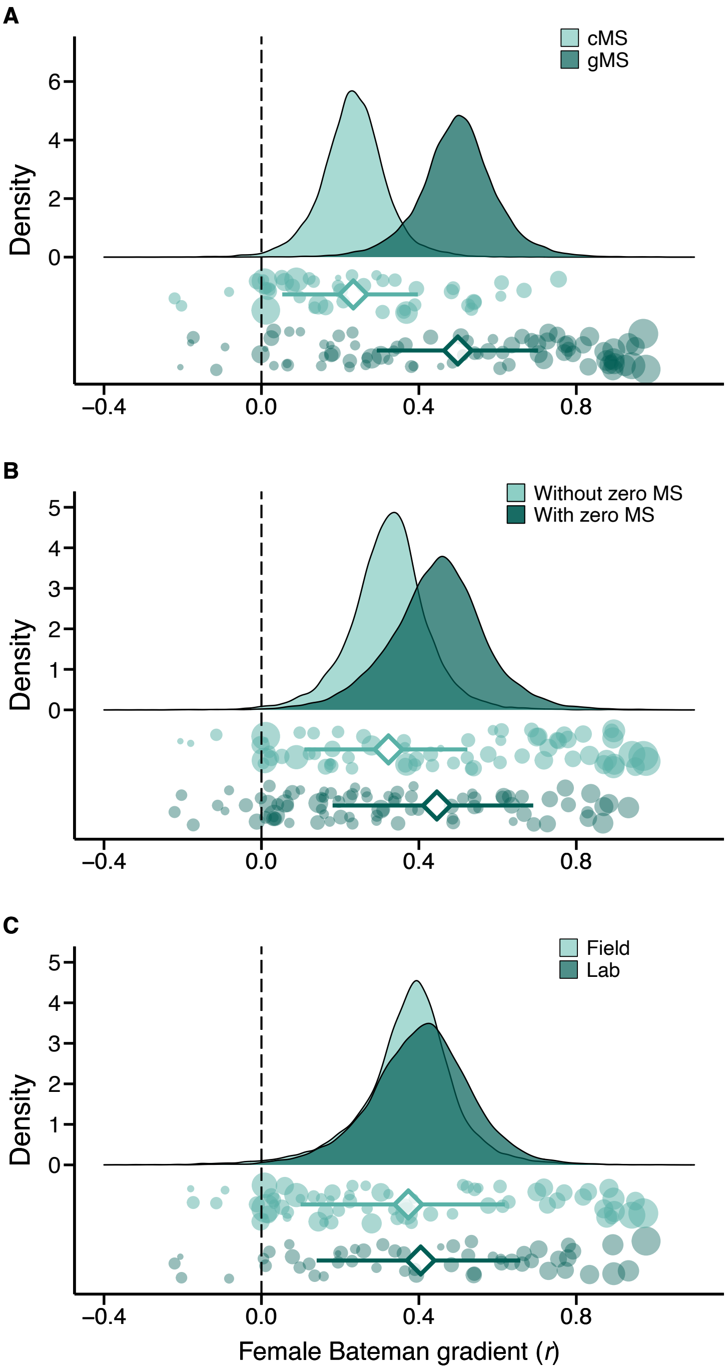

Figure B. Methodological predictors of female Bateman gradients. Raincloud charts showing effects of mating success method (*cMS*: copulatory mating success, *gMS*: genetic mating success), mating success range (with or without zero mating success (MS) category) and study type (field versus laboratory studies) on female Bateman gradients (see also Table 2 and Table C in S1 Text). The code and data needed to generate this figure can be found at https://salomefromonteil.github.io/META_SexSelFem/ and https://doi.org/10.5281/zenodo.7303598.

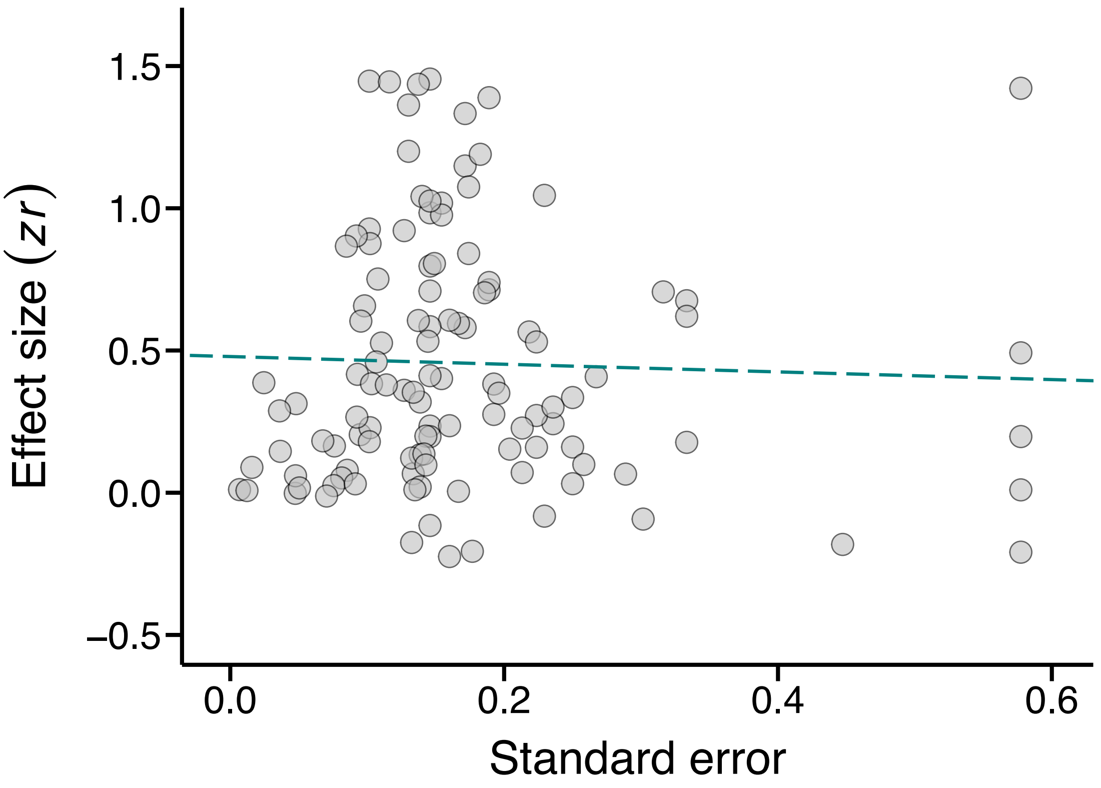

Figure C. Egger's regression. Relationship between effect size Fisher's *z* and its standard error testing whether small studies (i.e., those with smaller sample sizes and lower precision) are more likely to be published when reporting larger effect sizes. Dashed line shows the regression fit. The code and data needed to generate this figure can be found at https://salomefromonteil.github.io/META_SexSelFem/ and https://doi.org/10.5281/zenodo.7303598.

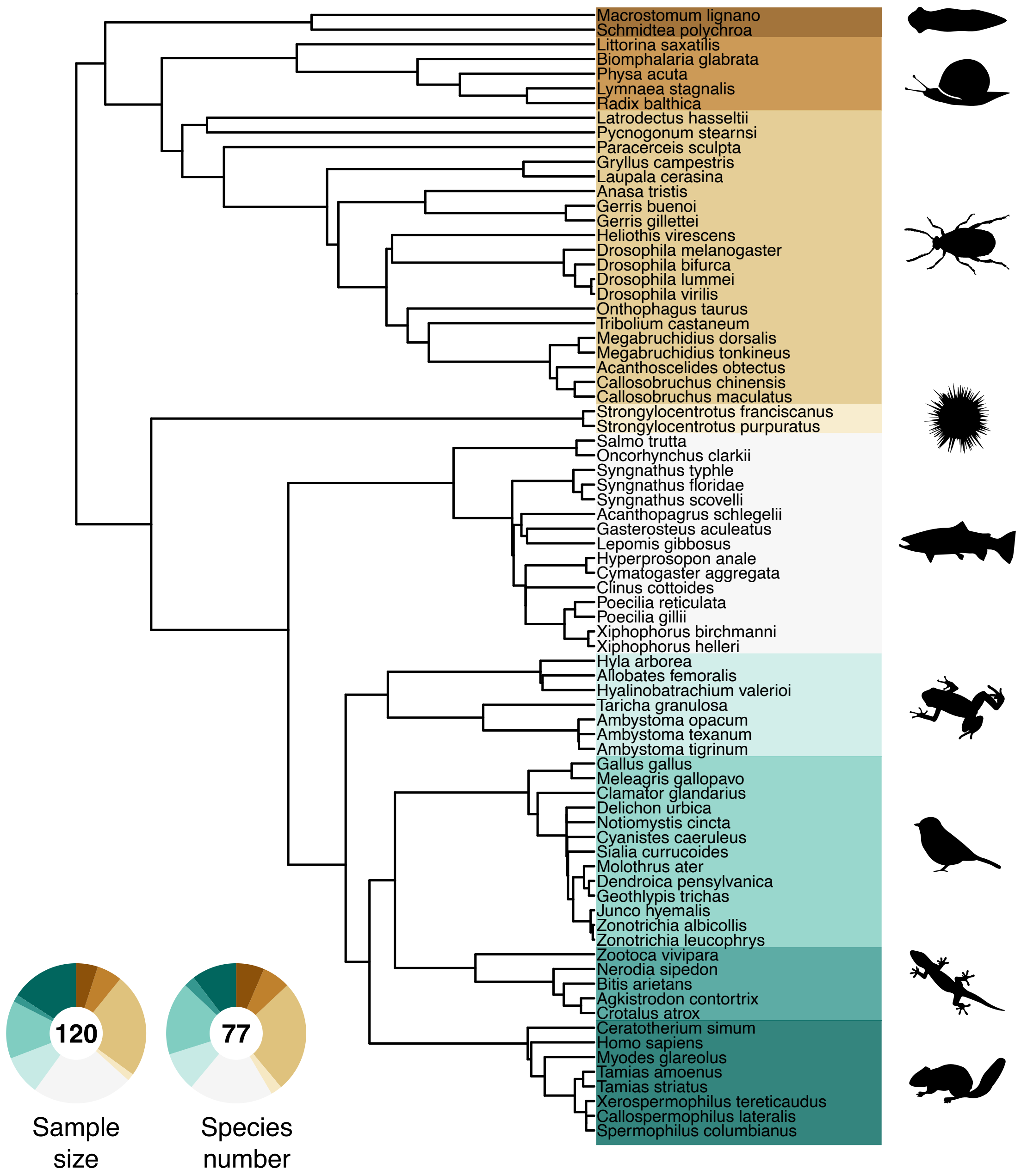

Figure D. Phylogenetic tree of all sampled species. Doughnut charts show the relative fraction of the sampled effect sizes (i.e., number of Bateman gradients) and the number of species.

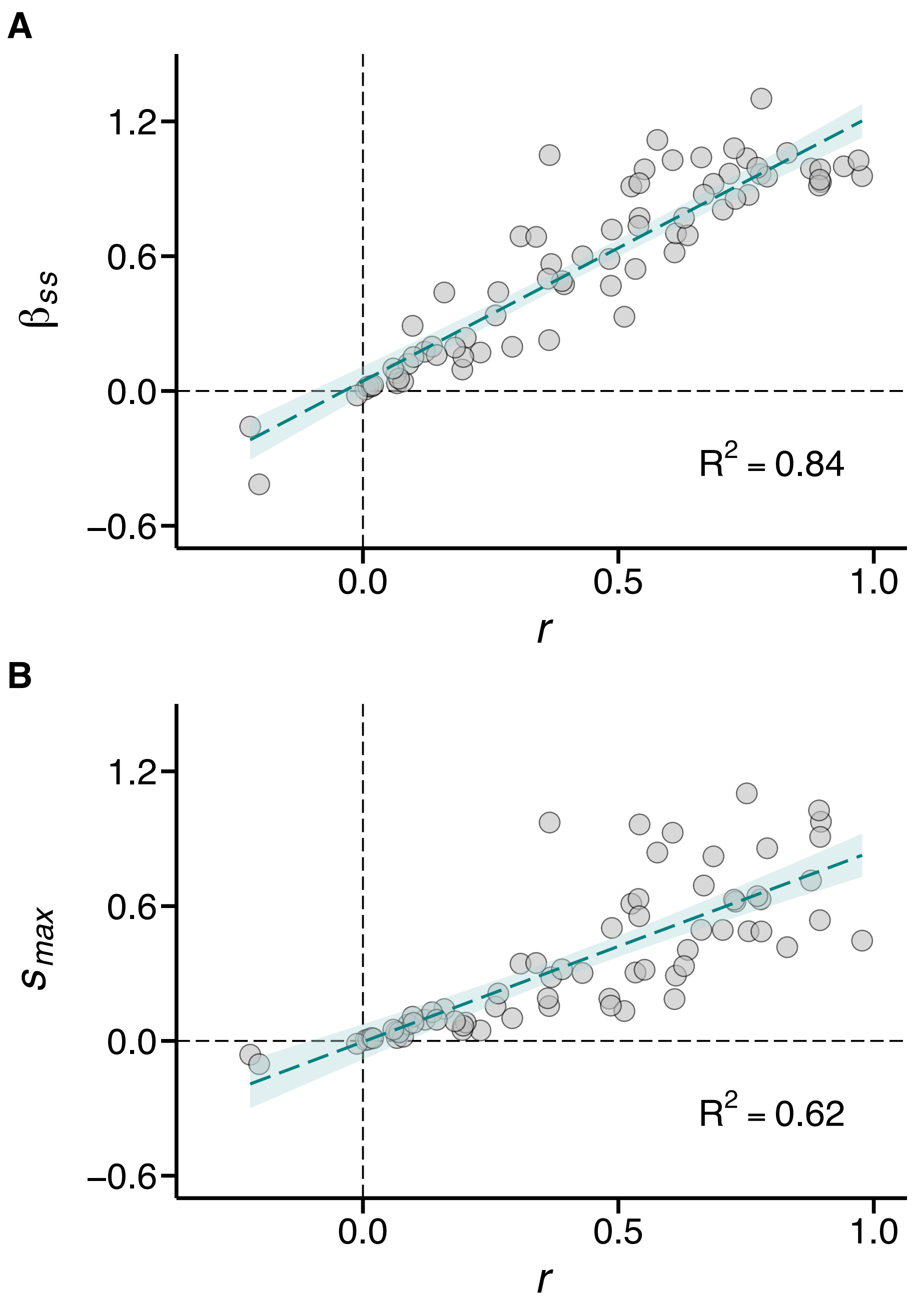

Figure E. Significance of used effect size. Relationship between effect size *r* (correlation coefficient) and (A) the actual Bateman gradient (β_{SS}) and (B) the maximum standardized sexual selection differential (s'_{max}). Graphs show a subset of data for which (A) standardized Bateman gradients were available (*N* = 74) and (B) estimates of the variance in relativized mating success could be extracted from primary studies (*N* = 73). Dashed line shows the linear regression fit with shaded area indicating the 95% confidence intervals. The code and data needed to generate this figure can be found at https://salomefromonteil.github.io/META_SexSelFem/ and https://doi.org/10.5281/zenodo.7303598.

Table A. Global tests of sexual selection in females using the restricted maximum likelihood (REML) approach. Results of intercept-only phylogenetically controlled General Linear-Mixed Effects Models are shown for the entire dataset (global model) and subsets with respect to mating success method (copulatory versus genetic), mating success range (including versus excluding zero mating success category), study type (laboratory versus field studies) and mating system (low-polyandry versus high-polyandry species). Table shows number of effect sizes (*k*), number of species (*N*) and estimates of *r* together with 95% confidence intervals (in parentheses).

Model	*k*	N_{Species}	Global effect size			
			r		*z*-value	*P*-value
Global model	120	77	0.37	(0.15, 0.59)	3.33	0.001
Copulatory mating success	43	24	0.23	(0.06, 0.41)	2.66	0.008
Genetic mating success	79	56	0.50	(0.32, 0.68)	5.41	< 0.001
Including zero mating success	70	42	0.43	(0.18, 0.68)	3.34	0.001
Excluding zero mating success	79	58	0.32	(0.17, 0.48)	4.09	< 0.001
Laboratory studies	52	31	0.40	(0.15, 0.65)	3.10	0.002
Field studies	68	47	0.40	(0.31, 0.49)	8.96	< 0.001
High-polyandry species	32	16	0.23	(0.09, 0.37)	3.16	0.002
Low-polyandry species	88	61	0.41	(0.16, 0.66)	3.20	0.001

Table B. Comparison of Bateman gradients among major taxonomic groups. Results of intercept-only phylogenetically controlled General Linear-Mixed Effects Models are shown all classes for which at least 10 effect sizes have been obtained from the literature. Table shows number of effect sizes (*k*), number of species (*N*), and effect sizes (*r*) with posterior modes with 95% Highest Posterior Density (HPD) intervals in parentheses.

Taxonomic group	*k*	N_{Species}	Effect size		
			r		P_{MCMC}
Arthropoda	29	20	0.38	(0.25, 0.52)	< 0.001
Actinopterygii	28	15	0.59	(0.45, 0.73)	< 0.001
Amphibia	11	7	0.47	(0.21, 0.72)	0.005
Aves	16	13	0.37	(0.20, 0.51)	< 0.001
Mammalia	19	8	0.37	(0.15, 0.60)	0.004

Table C. Predictors of inter-specific variation in female Bateman gradients using restricted maximum likelihood (REML) approach. Methodological moderators include mating success method (copulatory versus genetic mating success), mating success range (including versus excluding mating success category), study type (field versus lab) and year of publication (continuous variable). Effect of mating system contrasts low-polyandry and high-polyandry species. Effect of polyandry (continuous variable) estimates the relationship between the female Bateman gradient and the proportion of polyandrous females in the population. Phylogenetically controlled multilevel meta-analytic single predictor models are shown with Omnibus tests (Wald-type chi-square test) and McFadden's R^2.

Moderator	Estimate ± SE	Q_M	P-value	R^2
Mating success method	0.28 ± 0.09	10.71	0.001	0.23
Mating success range	0.16 ± 0.06	6.48	0.011	0.14
Study type	0.07 ± 0.09	0.62	0.429	0.03
Year	0.00 ± 0.01	0.67	0.414	0.01
Mating system	0.31 ± 0.07	16.92	< 0.001	0.35
Polyandry	0.66 ± 0.14	23.30	< 0.001	0.47

Table D. Estimates of polyandry used to assess the mating system classification (low-polyandry and high-polyandry species). Table shows estimates of all 77 sampled species in alphabetical order together with reference.

Species	Polyandry	Reference
Acanthopagrus schlegelii	0.920	[1]
Acanthoscelides obtectus	0.551	*
Agkistrodon contortrix	0.520	[2]
Allobates femoralis	0.571	[3]
Ambystoma opacum	0.294	[4]
Ambystoma texanum	0.857	[5]
Ambystoma tigrinum	0.467	[6]
Anasa tristis	0.950	[7]
Biomphalaria glabrata	0.654	[8]
Bitis arietans	0.941	[9]
Callosobruchus chinensis	0.680	[10] ¶
Callosobruchus maculatus	1.000	[10] ¶
Callospermophilus lateralis	0.630	[11]
Ceratotherium simum	0.697	[12]
Clamator glandarius	0.308	[13]
Clinus cottoides	0.826	[14]
Crotalus atrox	0.400	[15]
Cyanistes caeruleus	0.470	[16]
Cymatogaster aggregata	0.800	[17]
Delichon urbica	0.235	[18]
Dendroica pensylvanica	0.606	[19]
Drosophila bifurca	0.326	[20] ¶¶
Drosophila lummei	0.753	[21] ¶¶
Drosophila melanogaster	0.759	[22]
Drosophila virilis	0.753	[21] ¶¶
Gallus gallus	0.950	[23] ¶
Gasterosteus aculeatus	0.783	[24]
Geothlypis trichas	0.654	[25]
Gerris buenoi	1.000	[26]
Gerris gillettei	0.776	[27]
Gryllus campestris	0.809	[28]
Heliothis virescens	0.655	[29]
Homo sapiens	0.012	[30]
Hyalinobatrachium valerioi	0.736	[31]
Hyla arborea	0.158	[32]
Hyperprosopon anale	1.000	[17]
Junco hyemalis	0.439	[33]
Latrodectus hasselti	0.667	[34]
Laupala cerasina	1.000	[35]
Lepomis gibbosus	0.244	[36]

Species	Polyandry	Reference
Littorina saxatilis	0.680	[37]
Lymnaea stagnalis	0.588	[38]
Macrostomum lignano	1.000	[39]
Megabruchidius dorsalis	1.000	[10] ¶
Megabruchidius tonkineus	0.977	[10] ¶
Meleagris gallopavo	0.450	[40]
Molothrus ater	0.455	[41]
Myodes glareolus	0.353	[42]
Nerodia sipedon	0.556	[43]
Notiomystis cincta	0.836	[44]
Oncorhynchus clarkii	0.552	[45]
Onthophagus taurus	0.789	[46]
Paracerceis sculpta	0.667	[47]
Physa acuta	0.789	[48]
Poecilia gillii	0.709	[49]
Poecilia reticulata	0.710	[50]
Pycnogonum stearnsi	0.686	[51]
Radix balthica	0.500	[52]
Salmo trutta	0.680	[53]
Schmidtea polychroa	0.918	[54]
Sialia currucoides	0.342	[55]
Spermophilus columbianus	0.343	[56]
Strongylocentrotus franciscanus	0.990	[57]
Strongylocentrotus purpuratus	0.990	[57]
Syngnathus floridae	0.633	[58]
Syngnathus scovelli	0.615	[59]
Syngnathus typhle	0.528	[60]
Tamias amoenus	0.595	[61]
Tamias striatus	0.650	[62]
Taricha granulosa	0.548	[63]
Tribolium castaneum	0.661	**
Xerospermophilus tereticaudus	0.935	[64]
Xiphophorus birchmanni	0.839	[32]
Xiphophorus helleri	0.638	[65]
Zonotrichia albicollis	0.306	[66]
Zonotrichia leucophrys	0.346	[67]
Zootoca vivipara	0.633	[68]

* Unpublished study by Fromonteil and colleagues.
** Unpublished study by Winkler and colleagues.
¶ Authors of primary studies provided on request additional data to compute polyandry.
¶¶ References could only be found for classification of the mating system. Polyandry was extrapolated from the average level of polyandry observed in low-polyandry and high-polyandry species, respectively.

References

1. Wang X, Liu S, Yang YQ, Wu LN, Huang WH, Wu RX, et al. Genetic evidence for the mating system and reproductive success of black sea bream (*Acanthopagrus schlegelii*). Ecol Evol. 2020;10(10):4483-94. doi: 10.1002/ece3.6215.
2. Levine BA, Smith CF, Schuett GW, Douglas MR, Davis MA, Douglas ME. Bateman-Trivers in the 21st Century: sexual selection in a North American pitviper. Biol J Linnean Soc. 2015;114(2):436-45. doi: 10.1111/bij.12434.
3. Ursprung E, Ringler M, Jehle R, Hodl W. Strong male/male competition allows for nonchoosy females: high levels of polygynandry in a territorial frog with paternal care. Mol Ecol. 2011;20(8):1759-71. doi: 10.1111/j.1365-294X.2011.05056.x.
4. Croshaw DA. Quantifying sexual selection: a comparison of competing indices with mating system data from a terrestrially breeding salamander. Biol J Linn Soc. 2010;99(1):73-83.
5. Gopurenko D, Williams RN, DeWoody JA. Reproductive and mating success in the small-mouthed salamander (*Ambystoma texanum*) estimated via microsatellite parentage analysis. Evolutionary Biology. 2007;34(3-4):130-9. doi: 10.1007/s11692-007-9009-0.
6. Gopurenko D, Williams RN, McCormick CR, DeWoody JA. Insights into the mating habits of the tiger salamander (Ambystoma tigrinum tigrinum) as revealed by genetic parentage analyses. Mol Ecol. 2006;15(7):1917-28. doi: 10.1111/j.1365-294X.2006.02904.x.
7. Greenway EV, Hamel JA, Miller CW. Exploring the effects of extreme polyandry on estimates of sexual selection and reproductive success. Behavioral Ecology. 2021;32(6):1055-63. doi: 10.1093/beheco/arab081.
8. Anthes N, David P, Auld JR, Hoffer JN, Jarne P, Koene JM, et al. Bateman gradients in hermaphrodites: an extended approach to quantify sexual selection. Am Nat. 2010;176(3):249-63.
9. Glaudas X, Rice SE, Clark RW, Alexander GJ. The intensity of sexual selection, body size and reproductive success in a mating system with male-male combat: is bigger better? Oikos. 2020;129(7):998-1011. doi: 10.1111/oik.07223.
10. Fritzsche K, Arnqvist G. Homage to Bateman: sex roles predict sex differences in sexual selection. Evolution. 2013;67(7):1926-36. Epub 2013/07/03. doi: 10.1111/evo.12086.
11. Wells CP, Tomalty KM, Floyd CH, McElreath MB, May BP, Van Vuren DH. Determinants of multiple paternity in a fluctuating population of ground squirrels. Behav Ecol Sociobiol. 2017;71(2):13. doi: 10.1007/s00265-017-2270-z.
12. Kretzschmar P, Auld H, Boag P, Ganslosser U, Scott C, de Groot PJV, et al. Mate choice, reproductive success and inbreeding in white rhinoceros: New insights for conservation management. Evol Appl. 2020;13(4):699-714. doi: 10.1111/eva.12894.
13. Bolopo D, Canestrari D, Martinez JG, Roldan M, Macias-Sanchez E, Vila M, et al. Flexible mating patterns in an obligate brood parasite. Ibis. 2017;159(1):103-12. doi: 10.1111/ibi.12429.
14. Scheepers MJ, Gouws G. Mating system, reproductive success, and sexual selection in Bluntnose Klipfishes (*Clinus cottoides*). J Hered. 2019;110(3):351-60. doi: 10.1093/jhered/esz008.
15. Levine BA, Schuett GW, Clark RW, Repp RA, Herrmann HW, Booth W. No evidence of male-biased sexual selection in a snake with conventional Darwinian sex roles. R Soc Open Sci. 2020;7(10):10. doi: 10.1098/rsos.201261.

16. Schlicht E, Kempenaers B. Effects of social and extra-pair mating on sexual selection in blue tits (*Cyanistes caeruleus*) Evolution. 2013;67(5):1420-34. doi: 10.1111/evo.12073.

17. LaBrecque JR, Alva-Campbell YR, Archambeault S, Crow KD. Multiple paternity is a shared reproductive strategy in the live-bearing surfperches (Embiotocidae) that may be associated with female fitness. Ecology and Evolution. 2014;4(12):2316-29. doi: 10.1002/ece3.1071.

18. Whittingham LA, Lifjeld JT. High paternal investment in unrelated young: extra-pair paternity and male parental care in house martins. Behav Ecol Sociobiol. 1995;37(2):103-8. doi: 10.1007/s0026550370103.

19. Byers BE, Mays HL, Stewart IRK, Westneat DF. Extrapair paternity increases variability in male reproductive success in the chestnut-slded warbler (*Dendroica pensylvanica*), a socially monogamous songbird. Auk. 2004;121(3):788-95. doi: 10.1642/0004-8038(2004)121[0788:Epivim]2.0.Co;2.

20. Mery F, Joly D. Multiple mating, sperm transfer and oviposition pattern in the giant sperm species, Drosophila bifurca. J Evol Biol. 2002;15(1):49-56.

21. Bjork A, Pitnick S. Intensity of sexual selection along the anisogamy-isogamy continuum. Nature. 2006;441(7094):742-5.

22. Morimoto J, Pizzari T, Wigby S. Developmental environment effects on sexual selection in male and female *Drosophila melanogaster*. PLoS One. 2016;11(5):27. doi: 10.1371/journal.pone.0154468.

23. Collet J, Richardson DS, Worley K, Pizzari T. Sexual selection and the differential effect of polyandry. Proc Natl Acad Sci U S A. 2012;109(22):8641-5. doi: 10.1073/pnas.1200219109.

24. Fuxjager L, Wanzenbock S, Ringler E, Wegner KM, Ahnelt H, Shama LNS. Within-generation and transgenerational plasticity of mate choice in oceanic stickleback under climate change. Philos Trans R Soc B-Biol Sci. 2019;374(1768):12. doi: 10.1098/rstb.2018.0183.

25. Whittingham LA, Dunn PO. Effects of extra-pair and within-pair reproductive success on the opportunity for selection in birds. Behavioral Ecology. 2005;16(1):138-44. doi: 10.1093/beheco/arh140.

26. Devost E, Turgeon J. The combined effects of pre- and post-copulatory processes are masking sexual conflict over mating rate in Gerris buenoi. J Evol Biol. 2016;29(1):167-77. doi: 10.1111/jeb.12772.

27. Gagnon M-C, Duchesne P, Turgeon J. Sexual conflict in *Gerris gillettei* (Insecta: Hemiptera): influence of effective mating rate and morphology on reproductive success. Canadian Journal of Zoology. 2012;90(11):1297-306. doi: 10.1139/z2012-098.

28. Rost R, Honegger HW. The timing of premating and mating behavior in a field population of the cricket *Gryllus campestris* L. Behav Ecol Sociobiol. 1987;21(5):279-89. doi: 10.1007/bf00299965.

29. Greeff JM, Michiels NK. Low potential for sexual selection in simultaneously hermaphroditic animals. Proc R Soc B-Biol Sci. 1999;266(1429):1671-6.

30. Larmuseau MHD, Matthijs K, Wenseleers T. Cuckolded fathers rare in human populations. Trends in Ecology & Evolution. 2016;31(5):327-9. doi: 10.1016/j.tree.2016.03.004.

31. Mangold A, Trenkwalder K, Ringler M, Hoedl W, Ringler E. Low reproductive skew despite high male-biased operational sex ratio in a glass frog with paternal care. Bmc Evolutionary Biology. 2015;15. doi: 10.1186/s12862-015-0469-z.

32. Paczolt KA, Passow CN, Delclos PJ, Kindsvater HK, Jones AMG, Rosenthal GG. Multiple mating and reproductive skew in parental and introgressed females of the live-bearing fish *Xiphophorus birchmanni*. J Hered. 2015;106(1):57-66. doi: 10.1093/jhered/esu066.

33. Ketterson ED, Parker PG, Raouf SA, Nolan Jr V, Ziegenfus C, Chandler CH. The relative impact of extra-pair fertilizations on variation in male and female reproductive success in dark-eyed juncos (*Junco hyemaus*). In: Parker PG, Burley NT, editors. Avian Reproductive Tactics: Female and Male Perspectives. 491997. p. 81-101.

34. Andrade MCB, Kasumovic MM. Terminal investment strategies and male mate choice: Extreme tests of Bateman. Integr Comp Biol. 2005;45(5):838-47. doi: 10.1093/icb/45.5.838.

35. Turnell BR, Shaw KL. High opportunity for postcopulatory sexual selection under field conditions. Evolution. 2015;69(8):2094-104. doi: 10.1111/evo.12721.

36. Rios-Cardenas O. Patterns of parental investment and sexual selection in teleost fishes: Do they support Bateman's principles? Integr Comp Biol. 2005;45(5):885-94. doi: 10.1093/icb/45.5.885.

37. Johannesson K, Saltin SH, Charrier G, Ring AK, Kvarnemo C, Andre C, et al. Non-random paternity of offspring in a highly promiscuous marine snail suggests postcopulatory sexual selection. Behav Ecol Sociobiol. 2016;70(8):1357-66. doi: 10.1007/s00265-016-2143-x.

38. Nakadera Y, Marien J, Van Straalen NM, Koene JM. Multiple mating in natural populations of a simultaneous hermaphrodite, *Lymnaea stagnalis*. Journal of Molluscan Studies. 2017;83:56-62. doi: 10.1093/mollus/eyw043.

39. Marie-Orleach L, Janicke T, Vizoso DB, David P, Scharer L. Quantifying episodes of sexual selection: Insights from a transparent worm with fluorescent sperm. Evolution. 2016;70(2):314-28. doi: 10.1111/evo.12861.

40. Krakauer AH. Sexual selection and the genetic mating system of Wild Turkeys. Condor. 2008;110(1):1-12. doi: 10.1525/cond.2008.110.1.1.

41. Strausberger BM, Ashley MV. Breeding biology of brood parasitic brown-headed cowbirds (*Molothrus ater*) characterized by parent-offspring and sibling-group reconstruction. Auk. 2003;120(2):433-45. doi: 10.1642/0004-8038(2003)120[0433:Bbobpb]2.0.Co;2.

42. Ratkiewicz M, Borkowska A. Multiple paternity in the bank vole (*Clethrionomys glareolus*): field and experimental data. Zeitschrift Fur Saugetierkunde-International Journal of Mammalian Biology. 2000;65(1):6-14.

43. Prosser MR, Weatherhead PJ, Gibbs HL, Brown GP. Genetic analysis of the mating system and opportunity for sexual selection in northern water snakes (*Nerodia sipedon*). Behavioral Ecology. 2002;13(6):800-7. doi: 10.1093/beheco/13.6.800.

44. Walker LK, Ewen JG, Brekke P, Kilner RM. Sexually selected dichromatism in the hihi *Notiomystis cincta*: multiple colours for multiple receivers. Journal of Evolutionary Biology. 2014;27(8):1522-35. doi: 10.1111/jeb.12417.

45. Hargrove JS, McCane J, Roth CJ, High B, Campbell MR. Mating systems and predictors of relative reproductive success in a Cutthroat Trout subspecies of conservation concern. Ecol Evol. 2021;11(16):11295-309. doi: 10.1002/ece3.7914.

46. McCullough EL, Buzatto BA, Simmons LW. Population density mediates the interaction between pre- and postmating sexual selection. Evolution. 2018;72(4):893-905. doi: 10.1111/evo.13455.

47. Saunders KM, Shuster SM. Bateman gradients and alternative mating strategies in a marine isopod. IntechOpen. 2019;(DOI: 10.5772/intechopen.88956). doi: DOI: 10.5772/intechopen.88956.

48. Pélissié B, Jarne P, David P. Sexual selection without sexual dimorphism: Bateman gradients in a simultaneous hermaphrodite. Evolution. 2012;66(1):66-81. Epub 2012/01/10. doi: 10.1111/j.1558-5646.2011.01442.x.

49. Dekker ML, Hagmayer A, Leon-Kloosterziel KM, Furness AI, Pollux BJA. High Degree of Multiple Paternity and Reproductive Skew in the Highly Fecund Live-Bearing FishPoecilia gillii(Family Poeciliidae). Front Ecol Evol. 2020;8:14. doi: 10.3389/fevo.2020.579105.

50. Cattelan S, Evans JP, Garcia-Gonzalez F, Morbiato E, Pilastro A. Dietary stress increases the total opportunity for sexual selection and modifies selection on condition-dependent traits. Ecol Lett. 2020;23(3):447-56. doi: 10.1111/ele.13443.

51. Barreto FS, Avise JC. Quantitative measures of sexual selection reveal no evidence for sex-role reversal in a sea spider with prolonged paternal care. Proc R Soc B-Biol Sci. 2010;277(1696):2951-6. doi: 10.1098/rspb.2010.0311.

52. Burkli A, Jokela J. Increase in multiple paternity across the reproductive lifespan in a sperm-storing, hermaphroditic freshwater snail. Mol Ecol. 2017;26(19):5264-78. doi: 10.1111/mec.14200.

53. Largiader CR, Estoup A, Lecerf F, Champigneulle A, Guyomard R. Microsatellite analysis of polyandry and spawning site competition in brown trout (*Salmo trutta* L.). Genetics Selection Evolution. 2001;33:S205-S22. doi: 10.1186/bf03500881.

54. Pongratz N, Michiels NK. High multiple paternity and low last-male sperm precedence in a hermaphroditic planarian flatworm: consequences for reciprocity patterns. Mol Ecol. 2003;12(6):1425-33.

55. Brouwer L, Griffith SC. Extra-pair paternity in birds. Mol Ecol. 2019;28(22):4864-82. doi: 10.1111/mec.15259.

56. Jones PH, Van Zant JL, Dobson FS. Variation in reproductive success of male and female Columbian ground squirrels (Urocitellus columbianus). Canadian Journal of Zoology-Revue Canadienne De Zoologie. 2012;90(6):736-43. doi: 10.1139/z2012-042.

57. Levitan DR. Gamete traits influence the variance in reproductive success, the intensity of sexual selection, and the outcome of sexual conflict among congeneric sea urchins. Evolution. 2008;62(6):1305-16. doi: 10.1111/j.1558-5646.2008.00378.x.

58. Jones AG, Avise JC. Polygynandry in the dusky pipefish *Syngnathus floridae* revealed by microsatellite DNA markers. Evolution; international journal of organic evolution. 1997;51(5):1611-22. doi: 10.1111/j.1558-5646.1997.tb01484.x.

59. Jones AG, Walker D, Avise JC. Genetic evidence for extreme polyandry and extraordinary sex-role reversal in a pipefish. Proc Biol Sci. 2001;268(1485):2531-5. Epub 2001/12/26. doi: 10.1098/rspb.2001.1841.

60. Jones AG, Rosenqvist G, Berglund A, Avise JC. Mate quality influences multiple maternity in the sex-role-reversed pipefish *Syngnathus typhle*. Oikos. 2000;90(2):321-6. doi: 10.1034/j.1600-0706.2000.900212.x.

61. Schulte-Hostedde AI, Millar JS, Gibbs HL. Sexual selection and mating patterns in a mammal with female-biased sexual size dimorphism. Behavioral Ecology. 2004;15(2):351-6. doi: 10.1093/beheco/arh021.

62. Bergeron P, Montiglio PO, Reale D, Humphries MM, Garant D. Bateman gradients in a promiscuous mating system. Behav Ecol Sociobiol. 2012;66(8):1125-30. doi: 10.1007/s00265-012-1364-x.

63. Jones AG, Arguello JR, Arnold SJ. Molecular parentage analysis in experimental newt populations: The response of mating system measures to variation in the operational sex ratio. Am Nat. 2004;164(4):444-56. doi: 10.1086/423826.

64. Munroe KE, Koprowski JL. Sociality, Bateman's gradients, and the polygynandrous genetic mating system of round-tailed ground squirrels (*Xerospermophilus tereticaudus*). Behav Ecol Sociobiol. 2011;65(9):1811-24. doi: 10.1007/s00265-011-1189-z.

65. Tatarenkov A, Healey CIM, Grether GF, Avise JC. Pronounced reproductive skew in a natural population of green swordtails, *Xiphophorus helleri*. Mol Ecol. 2008;17(20):4522-34. doi: 10.1111/j.1365-294X.2008.03936.x.

66. Grunst AS, Grunst ML, Korody ML, Forrette LM, Gonser RA, Tuttle EM. Extrapair mating and the strength of sexual selection: insights from a polymorphic species. Behavioral Ecology. 2019;30(2):278-90. doi: 10.1093/beheco/ary160.

67. Poesel A, Gibbs HL, Nelson DA. Extrapair fertilizations and the potential for sexual selection in a socially monogamous songbird. Auk. 2011;128(4):770-6. doi: 10.1525/auk.2011.11127.

68. Laloi D, Richard M, Lecomte J, Massot M, Clobert J. Multiple paternity in clutches of common lizard *Lacerta vivipara*: data from microsatellite markers. Mol Ecol. 2004;13(3):719-23. doi: 10.1046/j.1365-294X.2004.02102.x.

Table E. Global tests of sexual selection in females excluding data on sex-role reversed species. Results of intercept-only phylogenetically controlled General Linear-Mixed Effects Models are shown for the dataset excluding sex-role reversed species (global model) and subsets with respect to mating success method (copulatory *versus* genetic), mating success range (including *versus* excluding zero mating success category), study type (laboratory *versus* field studies) and mating system (low-polyandry *versus* high-polyandry species). Table shows number of effect sizes (k), number of species (N), effect size (r), and heterogeneity I^2 arising from phylogenetic affinities, between-study variation, and between-observation variation. Model estimates are shown as posterior modes with 95% Highest Posterior Density (HPD) intervals in parentheses.

Model	k	$N_{Species}$	Effect size			Heterogeneity					
			r		P_{MCMC}	$I^2_{Phylogeny}$		I^2_{Study}		$I^2_{Observation}$	
Global model (non-phylogenetic)	109	71	0.38	(0.31, 0.45)	< 0.001	-		-		-	
Global model (phylogenetic)	109	71	0.35	(0.20, 0.49)	0.002	0.15	(0.00, 0.49)	0.66	(0.33, 0.91)	0.09	(0.00, 0.25)
Copulatory mating success	41	22	0.22	(0.08, 0.37)	0.008	0.30	(0.01, 0.72)	0.22	(0.00, 0.59)	0.24	(0.00, 0.63)
Genetic mating success	70	52	0.46	(0.29, 0.64)	0.001	0.16	(0.00, 0.51)	0.66	(0.28, 0.94)	0.09	(0.00, 0.28)
Including zero mating success	59	36	0.41	(0.22, 0.57)	0.002	0.20	(0.00, 0.63)	0.54	(0.08, 0.90)	0.13	(0.00, 0.39)
Excluding zero mating success	77	56	0.31	(0.12, 0.50)	0.007	0.24	(0.00, 0.67)	0.62	(0.21, 0.94)	0.07	(0.00, 0.20)
Laboratory studies	43	27	0.38	(0.18, 0.56)	0.002	0.27	(0.00, 0.74)	0.37	(0.00, 0.78)	0.18	(0.00, 0.55)
Field studies	66	45	0.31	(0.00, 0.56)	0.062	0.28	(0.00, 0.79)	0.58	(0.10, 0.93)	0.07	(0.00, 0.20)
Low-polyandry species	32	16	0.22	(-0.02, 0.45)	0.066	0.24	(0.00, 0.68)	0.53	(0.01, 0.90)	0.12	(0.00, 0.48)
High-polyandry species	77	55	0.38	(0.17, 0.58)	0.003	0.45	(0.07, 0.81)	0.28	(0.00, 0.64)	0.14	(0.00, 0.37)

Table F. Predictors of inter-specific variation in female Bateman gradients excluding data on sex-role reversed species. Methodological moderators include mating success method (copulatory *versus* genetic mating success), mating success range (including *versus* excluding zero mating success category), study type (field *versus* lab) and year of publication (continuous variable). Effect of mating system contrasts low-polyandry and high-polyandry species. Effect of polyandry estimates the relationship between the female Bateman gradient and the proportion of polyandrous females in the population. Model estimates (i.e., estimated difference between groups) are shown as posterior modes with 95% Highest Posterior Density (HPD) intervals obtained from phylogenetically controlled General Linear-Mixed Effects Models. The variance explained by the moderator variable is given as the marginal R^2 with 95% HPD intervals in parentheses.

Moderator	Estimate		P_{MCMC}	R^2	
Mating success method	0.31	(0.17, 0.46)	< 0.001	0.22	(0.16, 0.29)
Mating success range	0.09	(0.02, 0.17)	0.016	0.02	(0.01, 0.03)
Study type	0.04	(-0.14, 0.23)	0.675	0.00	(0.00, 0.01)
Year of publication	0.00	(-0.01, 0.01)	0.729	0.00	(0.00, 0.00)
Mating system	0.28	(0.12, 0.43)	0.001	0.16	(0.10, 0.22)
Polyandry	0.62	(0.32, 0.91)	< 0.001	0.23	(0.14, 0.32)

Table G. Global tests of sexual selection in females excluding data on humans. Results of intercept-only phylogenetically controlled General Linear-Mixed Effects Models are shown for the dataset including all species except humans (global model) and subsets with respect to mating success method (copulatory *versus* genetic), mating success range (including *versus* excluding zero mating success category), study type (laboratory *versus* field studies) and mating system (low-polyandry *versus* high-polyandry species). Table shows number of effect sizes (*k*), number of species (*N*), effect size (*r*), and heterogeneity I^2 arising from phylogenetic affinities, between-study variation, and between-observation variation. Model estimates are shown as posterior modes with 95% Highest Posterior Density (HPD) intervals in parentheses.

Model	*k*	$N_{Species}$	Effect size			Heterogeneity					
			r		P_{MCMC}	$I^2_{Phylogeny}$		I^2_{Study}		$I^2_{Observation}$	
Global model (non-phylogenetic)	110	76	0.44	(0.37, 0.51)	< 0.001	-		-		-	
Global model (phylogenetic)	110	76	0.39	(0.19, 0.58)	0.002	0.25	(0.00, 0.69)	0.49	(0.03, 0.74)	0.01	(0.00, 0.36)
Copulatory mating success	33	23	0.28	(0.11, 0.44)	0.009	0.02	(0.00, 0.66)	0.02	(0.00, 0.44)	0.02	(0.00, 0.77)
Genetic mating success	79	56	0.50	(0.29, 0.70)	0.001	0.01	(0.00, 0.67)	0.68	(0.16, 0.91)	0.01	(0.00, 0.25)
Including zero mating success	64	41	0.47	(0.26, 0.66)	< 0.001	0.01	(0.00, 0.74)	0.02	(0.00, 0.71)	0.01	(0.00, 0.46)
Excluding zero mating success	74	57	0.33	(0.10, 0.56)	0.012	0.47	(0.01, 0.79)	0.35	(0.00, 0.78)	0.01	(0.00, 0.27)
Laboratory studies	52	31	0.41	(0.13, 0.65)	0.007	0.72	(0.11, 0.92)	0.01	(0.00, 0.51)	0.01	(0.00, 0.41)
Field studies	58	46	0.41	(0.18, 0.61)	0.007	0.01	(0.00, 0.63)	0.71	(0.04, 0.89)	0.01	(0.00, 0.44)
Low-polyandry species	22	15	0.25	(0.00, 0.48)	0.056	0.02	(0.00, 0.56)	0.01	(0.00, 0.83)	0.01	(0.00, 0.78)
High-polyandry species	88	61	0.41	(0.15, 0.67)	0.005	0.75	(0.27, 0.92)	0.01	(0.00, 0.41)	0.01	(0.00, 0.31)

Table H. Predictors of inter-specific variation in female Bateman gradients excluding data on humans. Methodological moderators include mating success method (copulatory *versus* genetic mating success), mating success range (including *versus* excluding zero mating success category), study type (field *versus* lab) and year of publication (continuous variable). Effect of mating system contrasts low-polyandry and high-polyandry species. Effect of polyandry estimates the relationship between the female Bateman gradient and the proportion of polyandrous females in the population. Model estimates (i.e., estimated difference between groups) are shown as posterior modes with 95% Highest Posterior Density (HPD) intervals obtained from phylogenetically controlled General Linear-Mixed Effects Models. The variance explained by the moderator variable is given as the marginal R^2 with 95% HPD intervals in parentheses.

Moderator	Estimate		P_{MCMC}	R^2	
Mating success method	0.28	(0.09, 0.46)	0.003	0.16	(0.10, 0.21)
Mating success range	0.12	(0.04, 0.20)	0.002	0.03	(0.02, 0.05)
Study type	0.05	(-0.13, 0.22)	0.563	0.01	(0.00, 0.01)
Year of publication	0.00	(-0.02, 0.01)	0.528	0.00	(0.00, 0.01)
Mating system	0.27	(0.10, 0.43)	0.002	0.10	(0.05, 0.14)
Polyandry	0.60	(0.25, 0.94)	0.001	0.09	(0.05, 0.15)

Supplementary Information – S1 Text

Sexual selection in females and the evolution of polyandry

Salomé Fromonteil[1¶], Lucas Marie-Orleach[2,3¶], Lennart Winkler[4], Tim Janicke[1,4*]

[1] CEFE, Univ Montpellier, CNRS, EPHE, IRD, Montpellier, France.

[2] Natural History Museum, University of Oslo, Oslo, Norway.

[3] CNRS, Université de Rennes 1, ECOBIO (Écosystèmes, biodiversité, évolution) - UMR 6553, Rennes, France.

[4] Applied Zoology, TU Dresden, Dresden, Germany.

¶ Both authors contributed equally to this work.

* tim.janicke@cefe.cnrs.fr

This Supplementary Information file includes: Figures A - E

Tables A - H

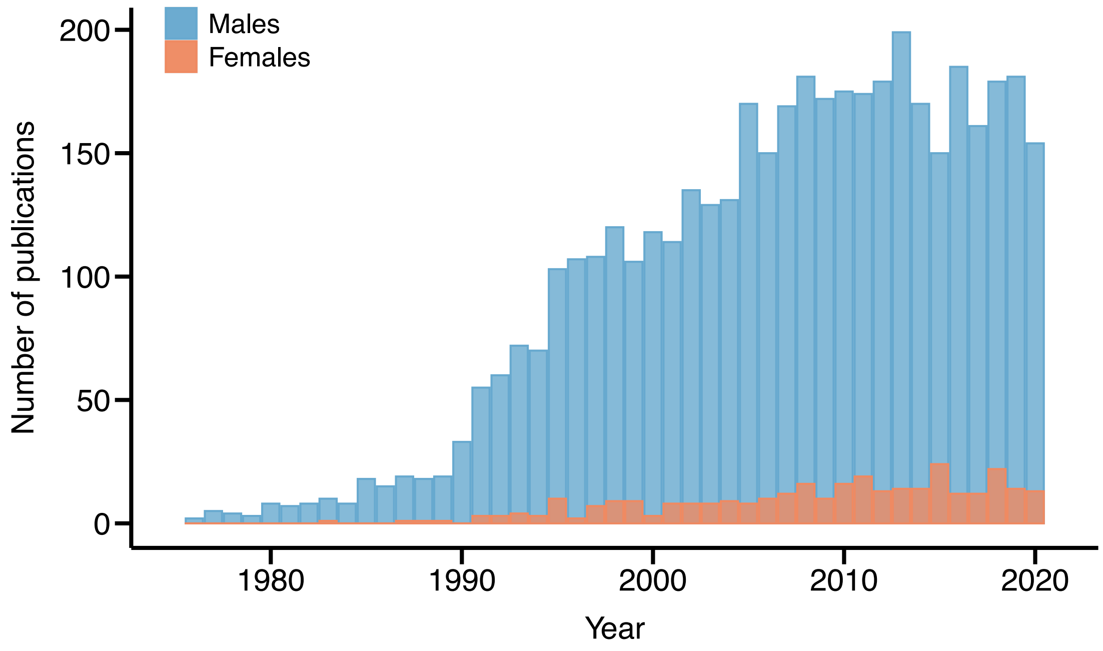

Figure A. Imbalance between studies of sexual selection in males and females. Bars indicate a strong male bias in the number of published articles on sexual selection indexed in ISI Web of Science (Clarivate Analytics) between 1900-2021. Data obtained from topic search using the search terms "sexual selection AND (male choice OR female competition)" for female and "sexual selection AND female choice OR male competition)" for male search. This is not meant to provide an exhaustive search of publications on sexual selection but to showcase the publication bias towards male studies focusing on Darwinian sexual selection in terms of competition for and choice of mating partners.

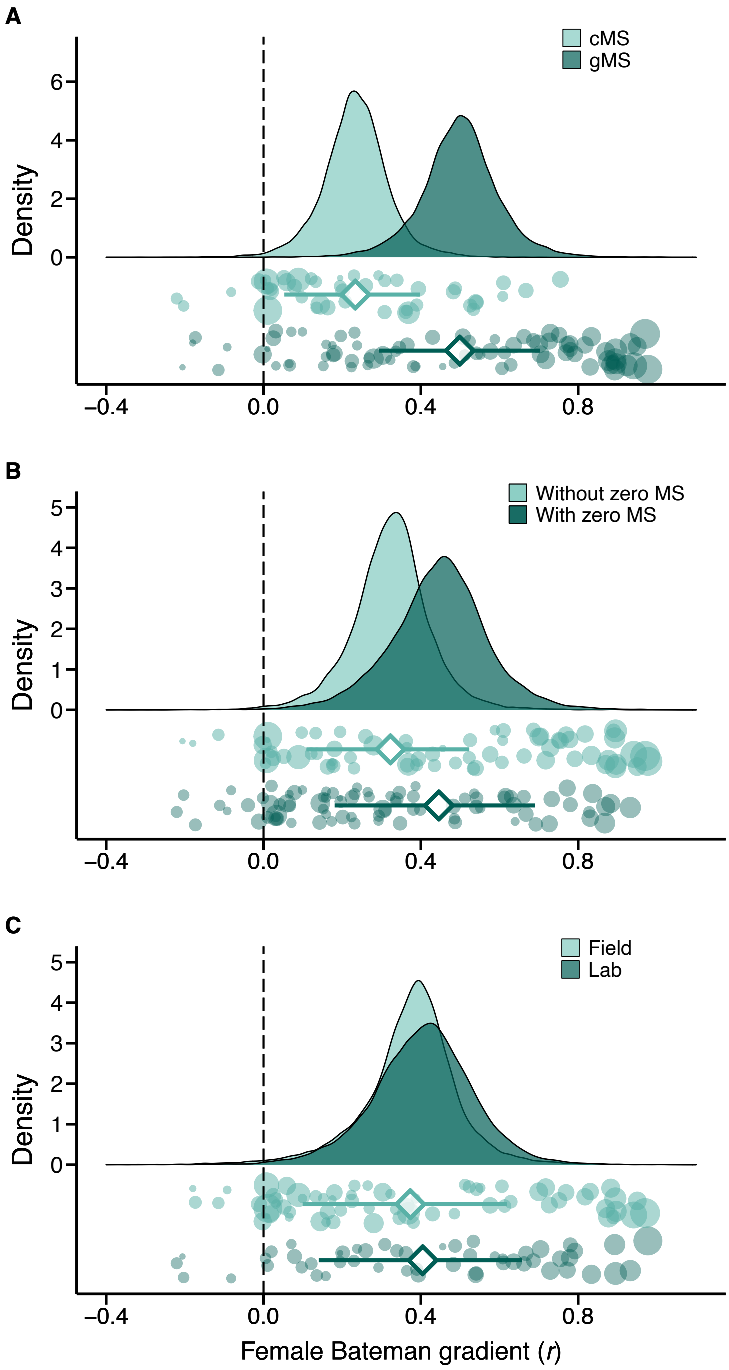

Figure B. Methodological predictors of female Bateman gradients. Raincloud charts showing effects of mating success method (*cMS*: copulatory mating success, *gMS*: genetic mating success), mating success range (with or without zero mating success (MS) category) and study type (field versus laboratory studies) on female Bateman gradients (see also Table 2 and Table C in S1 Text). The code and data needed to generate this figure can be found at https://salomefromonteil.github.io/META_SexSelFem/ and https://doi.org/10.5281/zenodo.7303598.

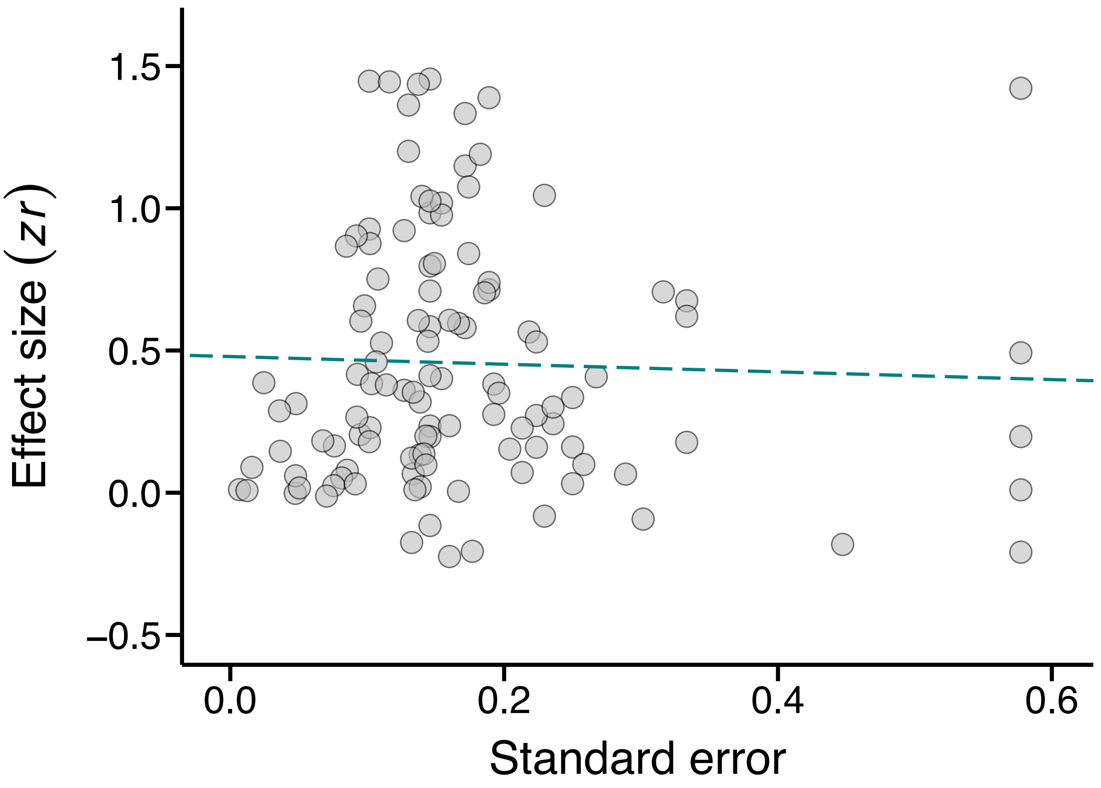

Figure C. Egger's regression. Relationship between effect size Fisher's *z* and its standard error testing whether small studies (i.e., those with smaller sample sizes and lower precision) are more likely to be published when reporting larger effect sizes. Dashed line shows the regression fit. The code and data needed to generate this figure can be found at https://salomefromonteil.github.io/META_SexSelFem/ and https://doi.org/10.5281/zenodo.7303598.

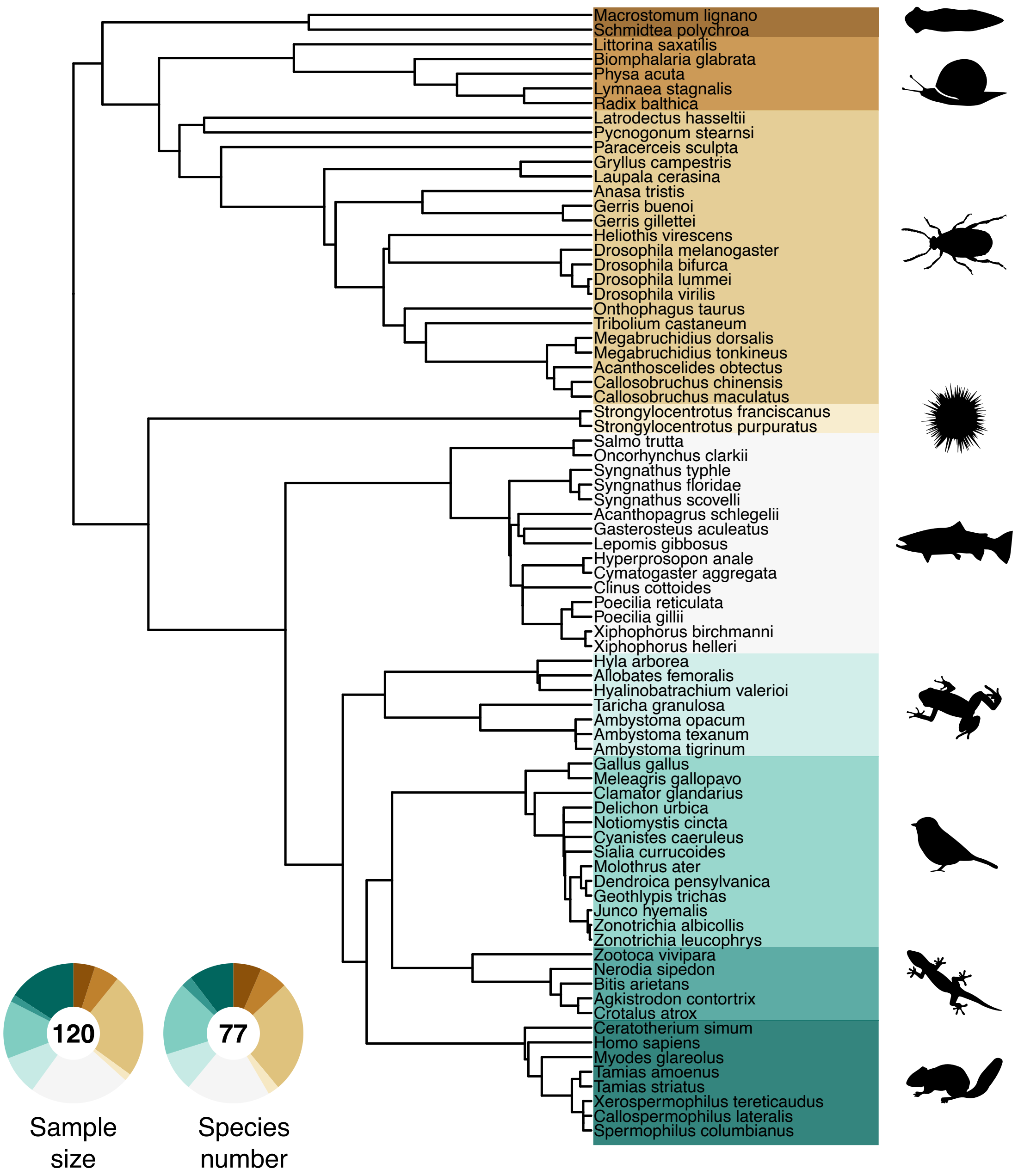

Figure D. Phylogenetic tree of all sampled species. Doughnut charts show the relative fraction of the sampled effect sizes (i.e., number of Bateman gradients) and the number of species.

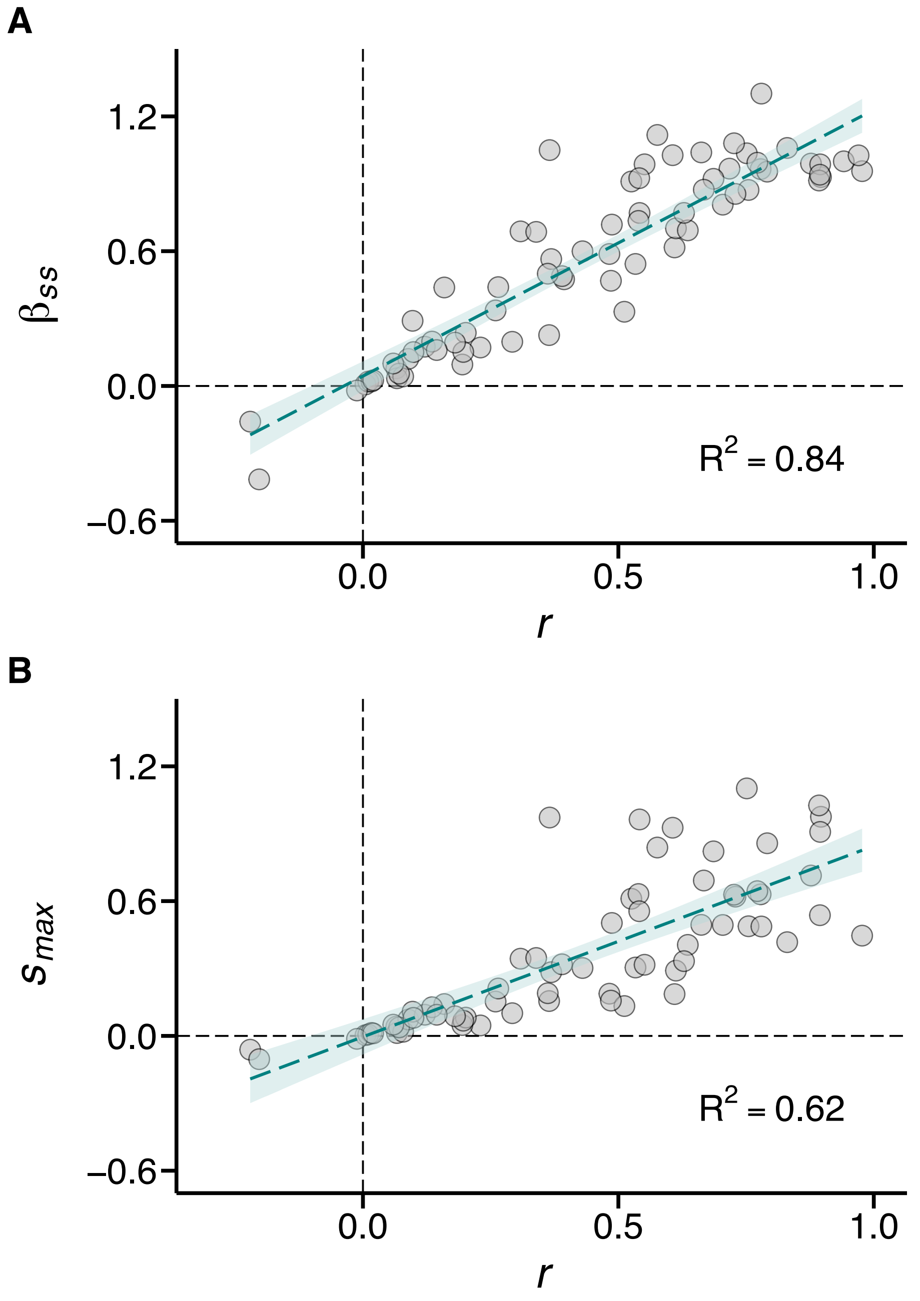

Figure E. Significance of used effect size. Relationship between effect size *r* (correlation coefficient) and (A) the actual Bateman gradient (β_{SS}) and (B) the maximum standardized sexual selection differential (s'_{max}). Graphs show a subset of data for which (A) standardized Bateman gradients were available ($N = 74$) and (B) estimates of the variance in relativized mating success could be extracted from primary studies ($N = 73$). Dashed line shows the linear regression fit with shaded area indicating the 95% confidence intervals. The code and data needed to generate this figure can be found at https://salomefromonteil.github.io/META_SexSelFem/ and https://doi.org/10.5281/zenodo.7303598.

Table A. Global tests of sexual selection in females using the restricted maximum likelihood (REML) approach. Results of intercept-only phylogenetically controlled General Linear-Mixed Effects Models are shown for the entire dataset (global model) and subsets with respect to mating success method (copulatory versus genetic), mating success range (including versus excluding zero mating success category), study type (laboratory versus field studies) and mating system (low-polyandry versus high-polyandry species). Table shows number of effect sizes (*k*), number of species (*N*) and estimates of *r* together with 95% confidence intervals (in parentheses).

Model	*k*	$N_{Species}$	Global effect size			
			r		*z*-value	*P*-value
Global model	120	77	0.37	(0.15, 0.59)	3.33	0.001
Copulatory mating success	43	24	0.23	(0.06, 0.41)	2.66	0.008
Genetic mating success	79	56	0.50	(0.32, 0.68)	5.41	< 0.001
Including zero mating success	70	42	0.43	(0.18, 0.68)	3.34	0.001
Excluding zero mating success	79	58	0.32	(0.17, 0.48)	4.09	< 0.001
Laboratory studies	52	31	0.40	(0.15, 0.65)	3.10	0.002
Field studies	68	47	0.40	(0.31, 0.49)	8.96	< 0.001
High-polyandry species	32	16	0.23	(0.09, 0.37)	3.16	0.002
Low-polyandry species	88	61	0.41	(0.16, 0.66)	3.20	0.001

Table B. Comparison of Bateman gradients among major taxonomic groups. Results of intercept-only phylogenetically controlled General Linear-Mixed Effects Models are shown all classes for which at least 10 effect sizes have been obtained from the literature. Table shows number of effect sizes (*k*), number of species (*N*), and effect sizes (*r*) with posterior modes with 95% Highest Posterior Density (HPD) intervals in parentheses.

Taxonomic group	k	N_{Species}	Effect size		
			r		P_{MCMC}
Arthropoda	29	20	0.38	(0.25, 0.52)	< 0.001
Actinopterygii	28	15	0.59	(0.45, 0.73)	< 0.001
Amphibia	11	7	0.47	(0.21, 0.72)	0.005
Aves	16	13	0.37	(0.20, 0.51)	< 0.001
Mammalia	19	8	0.37	(0.15, 0.60)	0.004

Table C. Predictors of inter-specific variation in female Bateman gradients using restricted maximum likelihood (REML) approach. Methodological moderators include mating success method (copulatory versus genetic mating success), mating success range (including versus excluding mating success category), study type (field versus lab) and year of publication (continuous variable). Effect of mating system contrasts low-polyandry and high-polyandry species. Effect of polyandry (continuous variable) estimates the relationship between the female Bateman gradient and the proportion of polyandrous females in the population. Phylogenetically controlled multilevel meta-analytic single predictor models are shown with Omnibus tests (Wald-type chi-square test) and McFadden's R^2.

Moderator	Estimate ± SE	Q_M	P-value	R^2
Mating success method	0.28 ± 0.09	10.71	0.001	0.23
Mating success range	0.16 ± 0.06	6.48	0.011	0.14
Study type	0.07 ± 0.09	0.62	0.429	0.03
Year	0.00 ± 0.01	0.67	0.414	0.01
Mating system	0.31 ± 0.07	16.92	< 0.001	0.35
Polyandry	0.66 ± 0.14	23.30	< 0.001	0.47

Table D. Estimates of polyandry used to assess the mating system classification (low-polyandry and high-polyandry species). Table shows estimates of all 77 sampled species in alphabetical order together with reference.

Species	Polyandry	Reference
Acanthopagrus schlegelii	0.920	[1]
Acanthoscelides obtectus	0.551	*
Agkistrodon contortrix	0.520	[2]
Allobates femoralis	0.571	[3]
Ambystoma opacum	0.294	[4]
Ambystoma texanum	0.857	[5]
Ambystoma tigrinum	0.467	[6]
Anasa tristis	0.950	[7]
Biomphalaria glabrata	0.654	[8]
Bitis arietans	0.941	[9]
Callosobruchus chinensis	0.680	[10] ¶
Callosobruchus maculatus	1.000	[10] ¶
Callospermophilus lateralis	0.630	[11]
Ceratotherium simum	0.697	[12]
Clamator glandarius	0.308	[13]
Clinus cottoides	0.826	[14]
Crotalus atrox	0.400	[15]
Cyanistes caeruleus	0.470	[16]
Cymatogaster aggregata	0.800	[17]
Delichon urbica	0.235	[18]
Dendroica pensylvanica	0.606	[19]
Drosophila bifurca	0.326	[20] ¶¶
Drosophila lummei	0.753	[21] ¶¶
Drosophila melanogaster	0.759	[22]
Drosophila virilis	0.753	[21] ¶¶
Gallus gallus	0.950	[23] ¶
Gasterosteus aculeatus	0.783	[24]
Geothlypis trichas	0.654	[25]
Gerris buenoi	1.000	[26]
Gerris gillettei	0.776	[27]
Gryllus campestris	0.809	[28]
Heliothis virescens	0.655	[29]
Homo sapiens	0.012	[30]
Hyalinobatrachium valerioi	0.736	[31]
Hyla arborea	0.158	[32]
Hyperprosopon anale	1.000	[17]
Junco hyemalis	0.439	[33]
Latrodectus hasselti	0.667	[34]
Laupala cerasina	1.000	[35]
Lepomis gibbosus	0.244	[36]

Species	Polyandry	Reference
Littorina saxatilis	0.680	[37]
Lymnaea stagnalis	0.588	[38]
Macrostomum lignano	1.000	[39]
Megabruchidius dorsalis	1.000	[10] ¶
Megabruchidius tonkineus	0.977	[10] ¶
Meleagris gallopavo	0.450	[40]
Molothrus ater	0.455	[41]
Myodes glareolus	0.353	[42]
Nerodia sipedon	0.556	[43]
Notiomystis cincta	0.836	[44]
Oncorhynchus clarkii	0.552	[45]
Onthophagus taurus	0.789	[46]
Paracerceis sculpta	0.667	[47]
Physa acuta	0.789	[48]
Poecilia gillii	0.709	[49]
Poecilia reticulata	0.710	[50]
Pycnogonum stearnsi	0.686	[51]
Radix balthica	0.500	[52]
Salmo trutta	0.680	[53]
Schmidtea polychroa	0.918	[54]
Sialia currucoides	0.342	[55]
Spermophilus columbianus	0.343	[56]
Strongylocentrotus franciscanus	0.990	[57]
Strongylocentrotus purpuratus	0.990	[57]
Syngnathus floridae	0.633	[58]
Syngnathus scovelli	0.615	[59]
Syngnathus typhle	0.528	[60]
Tamias amoenus	0.595	[61]
Tamias striatus	0.650	[62]
Taricha granulosa	0.548	[63]
Tribolium castaneum	0.661	**
Xerospermophilus tereticaudus	0.935	[64]
Xiphophorus birchmanni	0.839	[32]
Xiphophorus helleri	0.638	[65]
Zonotrichia albicollis	0.306	[66]
Zonotrichia leucophrys	0.346	[67]
Zootoca vivipara	0.633	[68]

* Unpublished study by Fromonteil and colleagues.
** Unpublished study by Winkler and colleagues.
¶ Authors of primary studies provided on request additional data to compute polyandry.
¶¶ References could only be found for classification of the mating system. Polyandry was extrapolated from the average level of polyandry observed in low-polyandry and high-polyandry species, respectively.

References

1. Wang X, Liu S, Yang YQ, Wu LN, Huang WH, Wu RX, et al. Genetic evidence for the mating system and reproductive success of black sea bream (*Acanthopagrus schlegelii*). Ecol Evol. 2020;10(10):4483-94. doi: 10.1002/ece3.6215.
2. Levine BA, Smith CF, Schuett GW, Douglas MR, Davis MA, Douglas ME. Bateman-Trivers in the 21st Century: sexual selection in a North American pitviper. Biol J Linnean Soc. 2015;114(2):436-45. doi: 10.1111/bij.12434.
3. Ursprung E, Ringler M, Jehle R, Hodl W. Strong male/male competition allows for nonchoosy females: high levels of polygynandry in a territorial frog with paternal care. Mol Ecol. 2011;20(8):1759-71. doi: 10.1111/j.1365-294X.2011.05056.x.
4. Croshaw DA. Quantifying sexual selection: a comparison of competing indices with mating system data from a terrestrially breeding salamander. Biol J Linn Soc. 2010;99(1):73-83.
5. Gopurenko D, Williams RN, DeWoody JA. Reproductive and mating success in the small-mouthed salamander (*Ambystoma texanum*) estimated via microsatellite parentage analysis. Evolutionary Biology. 2007;34(3-4):130-9. doi: 10.1007/s11692-007-9009-0.
6. Gopurenko D, Williams RN, McCormick CR, DeWoody JA. Insights into the mating habits of the tiger salamander (Ambystoma tigrinum tigrinum) as revealed by genetic parentage analyses. Mol Ecol. 2006;15(7):1917-28. doi: 10.1111/j.1365-294X.2006.02904.x.
7. Greenway EV, Hamel JA, Miller CW. Exploring the effects of extreme polyandry on estimates of sexual selection and reproductive success. Behavioral Ecology. 2021;32(6):1055-63. doi: 10.1093/beheco/arab081.
8. Anthes N, David P, Auld JR, Hoffer JN, Jarne P, Koene JM, et al. Bateman gradients in hermaphrodites: an extended approach to quantify sexual selection. Am Nat. 2010;176(3):249-63.
9. Glaudas X, Rice SE, Clark RW, Alexander GJ. The intensity of sexual selection, body size and reproductive success in a mating system with male-male combat: is bigger better? Oikos. 2020;129(7):998-1011. doi: 10.1111/oik.07223.
10. Fritzsche K, Arnqvist G. Homage to Bateman: sex roles predict sex differences in sexual selection. Evolution. 2013;67(7):1926-36. Epub 2013/07/03. doi: 10.1111/evo.12086.
11. Wells CP, Tomalty KM, Floyd CH, McElreath MB, May BP, Van Vuren DH. Determinants of multiple paternity in a fluctuating population of ground squirrels. Behav Ecol Sociobiol. 2017;71(2):13. doi: 10.1007/s00265-017-2270-z.
12. Kretzschmar P, Auld H, Boag P, Ganslosser U, Scott C, de Groot PJV, et al. Mate choice, reproductive success and inbreeding in white rhinoceros: New insights for conservation management. Evol Appl. 2020;13(4):699-714. doi: 10.1111/eva.12894.
13. Bolopo D, Canestrari D, Martinez JG, Roldan M, Macias-Sanchez E, Vila M, et al. Flexible mating patterns in an obligate brood parasite. Ibis. 2017;159(1):103-12. doi: 10.1111/ibi.12429.
14. Scheepers MJ, Gouws G. Mating system, reproductive success, and sexual selection in Bluntnose Klipfishes (*Clinus cottoides*). J Hered. 2019;110(3):351-60. doi: 10.1093/jhered/esz008.
15. Levine BA, Schuett GW, Clark RW, Repp RA, Herrmann HW, Booth W. No evidence of male-biased sexual selection in a snake with conventional Darwinian sex roles. R Soc Open Sci. 2020;7(10):10. doi: 10.1098/rsos.201261.

16. Schlicht E, Kempenaers B. Effects of social and extra-pair mating on sexual selection in blue tits (*Cyanistes caeruleus*) Evolution. 2013;67(5):1420-34. doi: 10.1111/evo.12073.

17. LaBrecque JR, Alva-Campbell YR, Archambeault S, Crow KD. Multiple paternity is a shared reproductive strategy in the live-bearing surfperches (Embiotocidae) that may be associated with female fitness. Ecology and Evolution. 2014;4(12):2316-29. doi: 10.1002/ece3.1071.

18. Whittingham LA, Lifjeld JT. High paternal investment in unrelated young: extra-pair paternity and male parental care in house martins. Behav Ecol Sociobiol. 1995;37(2):103-8. doi: 10.1007/s0026550370103.

19. Byers BE, Mays HL, Stewart IRK, Westneat DF. Extrapair paternity increases variability in male reproductive success in the chestnut-slded warbler (*Dendroica pensylvanica*), a socially monogamous songbird. Auk. 2004;121(3):788-95. doi: 10.1642/0004-8038(2004)121[0788:Epivim]2.0.Co;2.

20. Mery F, Joly D. Multiple mating, sperm transfer and oviposition pattern in the giant sperm species, Drosophila bifurca. J Evol Biol. 2002;15(1):49-56.

21. Bjork A, Pitnick S. Intensity of sexual selection along the anisogamy-isogamy continuum. Nature. 2006;441(7094):742-5.

22. Morimoto J, Pizzari T, Wigby S. Developmental environment effects on sexual selection in male and female *Drosophila melanogaster*. PLoS One. 2016;11(5):27. doi: 10.1371/journal.pone.0154468.

23. Collet J, Richardson DS, Worley K, Pizzari T. Sexual selection and the differential effect of polyandry. Proc Natl Acad Sci U S A. 2012;109(22):8641-5. doi: 10.1073/pnas.1200219109.

24. Fuxjager L, Wanzenbock S, Ringler E, Wegner KM, Ahnelt H, Shama LNS. Within-generation and transgenerational plasticity of mate choice in oceanic stickleback under climate change. Philos Trans R Soc B-Biol Sci. 2019;374(1768):12. doi: 10.1098/rstb.2018.0183.

25. Whittingham LA, Dunn PO. Effects of extra-pair and within-pair reproductive success on the opportunity for selection in birds. Behavioral Ecology. 2005;16(1):138-44. doi: 10.1093/beheco/arh140.

26. Devost E, Turgeon J. The combined effects of pre- and post-copulatory processes are masking sexual conflict over mating rate in Gerris buenoi. J Evol Biol. 2016;29(1):167-77. doi: 10.1111/jeb.12772.

27. Gagnon M-C, Duchesne P, Turgeon J. Sexual conflict in *Gerris gillettei* (Insecta: Hemiptera): influence of effective mating rate and morphology on reproductive success. Canadian Journal of Zoology. 2012;90(11):1297-306. doi: 10.1139/z2012-098.

28. Rost R, Honegger HW. The timing of premating and mating behavior in a field population of the cricket *Gryllus campestris* L. Behav Ecol Sociobiol. 1987;21(5):279-89. doi: 10.1007/bf00299965.

29. Greeff JM, Michiels NK. Low potential for sexual selection in simultaneously hermaphroditic animals. Proc R Soc B-Biol Sci. 1999;266(1429):1671-6.

30. Larmuseau MHD, Matthijs K, Wenseleers T. Cuckolded fathers rare in human populations. Trends in Ecology & Evolution. 2016;31(5):327-9. doi: 10.1016/j.tree.2016.03.004.

31. Mangold A, Trenkwalder K, Ringler M, Hoedl W, Ringler E. Low reproductive skew despite high male-biased operational sex ratio in a glass frog with paternal care. Bmc Evolutionary Biology. 2015;15. doi: 10.1186/s12862-015-0469-z.

32. Paczolt KA, Passow CN, Delclos PJ, Kindsvater HK, Jones AMG, Rosenthal GG. Multiple mating and reproductive skew in parental and introgressed females of the live-bearing fish *Xiphophorus birchmanni*. J Hered. 2015;106(1):57-66. doi: 10.1093/jhered/esu066.

33. Ketterson ED, Parker PG, Raouf SA, Nolan Jr V, Ziegenfus C, Chandler CH. The relative impact of extra-pair fertilizations on variation in male and female reproductive success in dark-eyed juncos (*Junco hyemaus*). In: Parker PG, Burley NT, editors. Avian Reproductive Tactics: Female and Male Perspectives. 491997. p. 81-101.

34. Andrade MCB, Kasumovic MM. Terminal investment strategies and male mate choice: Extreme tests of Bateman. Integr Comp Biol. 2005;45(5):838-47. doi: 10.1093/icb/45.5.838.

35. Turnell BR, Shaw KL. High opportunity for postcopulatory sexual selection under field conditions. Evolution. 2015;69(8):2094-104. doi: 10.1111/evo.12721.

36. Rios-Cardenas O. Patterns of parental investment and sexual selection in teleost fishes: Do they support Bateman's principles? Integr Comp Biol. 2005;45(5):885-94. doi: 10.1093/icb/45.5.885.

37. Johannesson K, Saltin SH, Charrier G, Ring AK, Kvarnemo C, Andre C, et al. Non-random paternity of offspring in a highly promiscuous marine snail suggests postcopulatory sexual selection. Behav Ecol Sociobiol. 2016;70(8):1357-66. doi: 10.1007/s00265-016-2143-x.

38. Nakadera Y, Marien J, Van Straalen NM, Koene JM. Multiple mating in natural populations of a simultaneous hermaphrodite, *Lymnaea stagnalis*. Journal of Molluscan Studies. 2017;83:56-62. doi: 10.1093/mollus/eyw043.

39. Marie-Orleach L, Janicke T, Vizoso DB, David P, Scharer L. Quantifying episodes of sexual selection: Insights from a transparent worm with fluorescent sperm. Evolution. 2016;70(2):314-28. doi: 10.1111/evo.12861.

40. Krakauer AH. Sexual selection and the genetic mating system of Wild Turkeys. Condor. 2008;110(1):1-12. doi: 10.1525/cond.2008.110.1.1.

41. Strausberger BM, Ashley MV. Breeding biology of brood parasitic brown-headed cowbirds (*Molothrus ater*) characterized by parent-offspring and sibling-group reconstruction. Auk. 2003;120(2):433-45. doi: 10.1642/0004-8038(2003)120[0433:Bbobpb]2.0.Co;2.

42. Ratkiewicz M, Borkowska A. Multiple paternity in the bank vole (*Clethrionomys glareolus*): field and experimental data. Zeitschrift Fur Saugetierkunde-International Journal of Mammalian Biology. 2000;65(1):6-14.

43. Prosser MR, Weatherhead PJ, Gibbs HL, Brown GP. Genetic analysis of the mating system and opportunity for sexual selection in northern water snakes (*Nerodia sipedon*). Behavioral Ecology. 2002;13(6):800-7. doi: 10.1093/beheco/13.6.800.

44. Walker LK, Ewen JG, Brekke P, Kilner RM. Sexually selected dichromatism in the hihi *Notiomystis cincta*: multiple colours for multiple receivers. Journal of Evolutionary Biology. 2014;27(8):1522-35. doi: 10.1111/jeb.12417.

45. Hargrove JS, McCane J, Roth CJ, High B, Campbell MR. Mating systems and predictors of relative reproductive success in a Cutthroat Trout subspecies of conservation concern. Ecol Evol. 2021;11(16):11295-309. doi: 10.1002/ece3.7914.

46. McCullough EL, Buzatto BA, Simmons LW. Population density mediates the interaction between pre- and postmating sexual selection. Evolution. 2018;72(4):893-905. doi: 10.1111/evo.13455.

47. Saunders KM, Shuster SM. Bateman gradients and alternative mating strategies in a marine isopod. IntechOpen. 2019;(DOI: 10.5772/intechopen.88956). doi: DOI: 10.5772/intechopen.88956.

48. Pélissié B, Jarne P, David P. Sexual selection without sexual dimorphism: Bateman gradients in a simultaneous hermaphrodite. Evolution. 2012;66(1):66-81. Epub 2012/01/10. doi: 10.1111/j.1558-5646.2011.01442.x.

49. Dekker ML, Hagmayer A, Leon-Kloosterziel KM, Furness AI, Pollux BJA. High Degree of Multiple Paternity and Reproductive Skew in the Highly Fecund Live-Bearing FishPoecilia gillii(Family Poeciliidae). Front Ecol Evol. 2020;8:14. doi: 10.3389/fevo.2020.579105.

50. Cattelan S, Evans JP, Garcia-Gonzalez F, Morbiato E, Pilastro A. Dietary stress increases the total opportunity for sexual selection and modifies selection on condition-dependent traits. Ecol Lett. 2020;23(3):447-56. doi: 10.1111/ele.13443.

51. Barreto FS, Avise JC. Quantitative measures of sexual selection reveal no evidence for sex-role reversal in a sea spider with prolonged paternal care. Proc R Soc B-Biol Sci. 2010;277(1696):2951-6. doi: 10.1098/rspb.2010.0311.

52. Burkli A, Jokela J. Increase in multiple paternity across the reproductive lifespan in a sperm-storing, hermaphroditic freshwater snail. Mol Ecol. 2017;26(19):5264-78. doi: 10.1111/mec.14200.

53. Largiader CR, Estoup A, Lecerf F, Champigneulle A, Guyomard R. Microsatellite analysis of polyandry and spawning site competition in brown trout (*Salmo trutta* L.). Genetics Selection Evolution. 2001;33:S205-S22. doi: 10.1186/bf03500881.

54. Pongratz N, Michiels NK. High multiple paternity and low last-male sperm precedence in a hermaphroditic planarian flatworm: consequences for reciprocity patterns. Mol Ecol. 2003;12(6):1425-33.

55. Brouwer L, Griffith SC. Extra-pair paternity in birds. Mol Ecol. 2019;28(22):4864-82. doi: 10.1111/mec.15259.

56. Jones PH, Van Zant JL, Dobson FS. Variation in reproductive success of male and female Columbian ground squirrels (Urocitellus columbianus). Canadian Journal of Zoology-Revue Canadienne De Zoologie. 2012;90(6):736-43. doi: 10.1139/z2012-042.

57. Levitan DR. Gamete traits influence the variance in reproductive success, the intensity of sexual selection, and the outcome of sexual conflict among congeneric sea urchins. Evolution. 2008;62(6):1305-16. doi: 10.1111/j.1558-5646.2008.00378.x.

58. Jones AG, Avise JC. Polygynandry in the dusky pipefish *Syngnathus floridae* revealed by microsatellite DNA markers. Evolution; international journal of organic evolution. 1997;51(5):1611-22. doi: 10.1111/j.1558-5646.1997.tb01484.x.

59. Jones AG, Walker D, Avise JC. Genetic evidence for extreme polyandry and extraordinary sex-role reversal in a pipefish. Proc Biol Sci. 2001;268(1485):2531-5. Epub 2001/12/26. doi: 10.1098/rspb.2001.1841.

60. Jones AG, Rosenqvist G, Berglund A, Avise JC. Mate quality influences multiple maternity in the sex-role-reversed pipefish *Syngnathus typhle*. Oikos. 2000;90(2):321-6. doi: 10.1034/j.1600-0706.2000.900212.x.

61. Schulte-Hostedde AI, Millar JS, Gibbs HL. Sexual selection and mating patterns in a mammal with female-biased sexual size dimorphism. Behavioral Ecology. 2004;15(2):351-6. doi: 10.1093/beheco/arh021.

62. Bergeron P, Montiglio PO, Reale D, Humphries MM, Garant D. Bateman gradients in a promiscuous mating system. Behav Ecol Sociobiol. 2012;66(8):1125-30. doi: 10.1007/s00265-012-1364-x.

63. Jones AG, Arguello JR, Arnold SJ. Molecular parentage analysis in experimental newt populations: The response of mating system measures to variation in the operational sex ratio. Am Nat. 2004;164(4):444-56. doi: 10.1086/423826.

64. Munroe KE, Koprowski JL. Sociality, Bateman's gradients, and the polygynandrous genetic mating system of round-tailed ground squirrels (*Xerospermophilus tereticaudus*). Behav Ecol Sociobiol. 2011;65(9):1811-24. doi: 10.1007/s00265-011-1189-z.

65. Tatarenkov A, Healey CIM, Grether GF, Avise JC. Pronounced reproductive skew in a natural population of green swordtails, *Xiphophorus helleri*. Mol Ecol. 2008;17(20):4522-34. doi: 10.1111/j.1365-294X.2008.03936.x.

66. Grunst AS, Grunst ML, Korody ML, Forrette LM, Gonser RA, Tuttle EM. Extrapair mating and the strength of sexual selection: insights from a polymorphic species. Behavioral Ecology. 2019;30(2):278-90. doi: 10.1093/beheco/ary160.

67. Poesel A, Gibbs HL, Nelson DA. Extrapair fertilizations and the potential for sexual selection in a socially monogamous songbird. Auk. 2011;128(4):770-6. doi: 10.1525/auk.2011.11127.

68. Laloi D, Richard M, Lecomte J, Massot M, Clobert J. Multiple paternity in clutches of common lizard *Lacerta vivipara*: data from microsatellite markers. Mol Ecol. 2004;13(3):719-23. doi: 10.1046/j.1365-294X.2004.02102.x.

Table E. Global tests of sexual selection in females excluding data on sex-role reversed species. Results of intercept-only phylogenetically controlled General Linear-Mixed Effects Models are shown for the dataset excluding sex-role reversed species (global model) and subsets with respect to mating success method (copulatory *versus* genetic), mating success range (including *versus* excluding zero mating success category), study type (laboratory *versus* field studies) and mating system (low-polyandry *versus* high-polyandry species). Table shows number of effect sizes (*k*), number of species (*N*), effect size (*r*), and heterogeneity I^2 arising from phylogenetic affinities, between-study variation, and between-observation variation. Model estimates are shown as posterior modes with 95% Highest Posterior Density (HPD) intervals in parentheses.

Model	k	N_{Species}	Effect size			Heterogeneity					
			r		P_{MCMC}	$I^2_{\text{Phylogeny}}$		I^2_{Study}		$I^2_{\text{Observation}}$	
Global model (non-phylogenetic)	109	71	0.38	(0.31, 0.45)	< 0.001	-		-		-	
Global model (phylogenetic)	109	71	0.35	(0.20, 0.49)	0.002	0.15	(0.00, 0.49)	0.66	(0.33, 0.91)	0.09	(0.00, 0.25)
Copulatory mating success	41	22	0.22	(0.08, 0.37)	0.008	0.30	(0.01, 0.72)	0.22	(0.00, 0.59)	0.24	(0.00, 0.63)
Genetic mating success	70	52	0.46	(0.29, 0.64)	0.001	0.16	(0.00, 0.51)	0.66	(0.28, 0.94)	0.09	(0.00, 0.28)
Including zero mating success	59	36	0.41	(0.22, 0.57)	0.002	0.20	(0.00, 0.63)	0.54	(0.08, 0.90)	0.13	(0.00, 0.39)
Excluding zero mating success	77	56	0.31	(0.12, 0.50)	0.007	0.24	(0.00, 0.67)	0.62	(0.21, 0.94)	0.07	(0.00, 0.20)
Laboratory studies	43	27	0.38	(0.18, 0.56)	0.002	0.27	(0.00, 0.74)	0.37	(0.00, 0.78)	0.18	(0.00, 0.55)
Field studies	66	45	0.31	(0.00, 0.56)	0.062	0.28	(0.00, 0.79)	0.58	(0.10, 0.93)	0.07	(0.00, 0.20)
Low-polyandry species	32	16	0.22	(-0.02, 0.45)	0.066	0.24	(0.00, 0.68)	0.53	(0.01, 0.90)	0.12	(0.00, 0.48)
High-polyandry species	77	55	0.38	(0.17, 0.58)	0.003	0.45	(0.07, 0.81)	0.28	(0.00, 0.64)	0.14	(0.00, 0.37)

Table F. Predictors of inter-specific variation in female Bateman gradients excluding data on sex-role reversed species. Methodological moderators include mating success method (copulatory *versus* genetic mating success), mating success range (including *versus* excluding zero mating success category), study type (field *versus* lab) and year of publication (continuous variable). Effect of mating system contrasts low-polyandry and high-polyandry species. Effect of polyandry estimates the relationship between the female Bateman gradient and the proportion of polyandrous females in the population. Model estimates (i.e., estimated difference between groups) are shown as posterior modes with 95% Highest Posterior Density (HPD) intervals obtained from phylogenetically controlled General Linear-Mixed Effects Models. The variance explained by the moderator variable is given as the marginal R^2 with 95% HPD intervals in parentheses.

Moderator	Estimate		P_{MCMC}	R^2	
Mating success method	0.31	(0.17, 0.46)	< 0.001	0.22	(0.16, 0.29)
Mating success range	0.09	(0.02, 0.17)	0.016	0.02	(0.01, 0.03)
Study type	0.04	(-0.14, 0.23)	0.675	0.00	(0.00, 0.01)
Year of publication	0.00	(-0.01, 0.01)	0.729	0.00	(0.00, 0.00)
Mating system	0.28	(0.12, 0.43)	0.001	0.16	(0.10, 0.22)
Polyandry	0.62	(0.32, 0.91)	< 0.001	0.23	(0.14, 0.32)

Table G. Global tests of sexual selection in females excluding data on humans. Results of intercept-only phylogenetically controlled General Linear-Mixed Effects Models are shown for the dataset including all species except humans (global model) and subsets with respect to mating success method (copulatory *versus* genetic), mating success range (including *versus* excluding zero mating success category), study type (laboratory *versus* field studies) and mating system (low-polyandry *versus* high-polyandry species). Table shows number of effect sizes (k), number of species (N), effect size (r), and heterogeneity I^2 arising from phylogenetic affinities, between-study variation, and between-observation variation. Model estimates are shown as posterior modes with 95% Highest Posterior Density (HPD) intervals in parentheses.

Model	k	$N_{Species}$	Effect size			Heterogeneity					
			r		P_{MCMC}	$I^2_{Phylogeny}$		I^2_{Study}		$I^2_{Observation}$	
Global model (non-phylogenetic)	110	76	0.44	(0.37, 0.51)	< 0.001	-		-		-	
Global model (phylogenetic)	110	76	0.39	(0.19, 0.58)	0.002	0.25	(0.00, 0.69)	0.49	(0.03, 0.74)	0.01	(0.00, 0.36)
Copulatory mating success	33	23	0.28	(0.11, 0.44)	0.009	0.02	(0.00, 0.66)	0.02	(0.00, 0.44)	0.02	(0.00, 0.77)
Genetic mating success	79	56	0.50	(0.29, 0.70)	0.001	0.01	(0.00, 0.67)	0.68	(0.16, 0.91)	0.01	(0.00, 0.25)
Including zero mating success	64	41	0.47	(0.26, 0.66)	< 0.001	0.01	(0.00, 0.74)	0.02	(0.00, 0.71)	0.01	(0.00, 0.46)
Excluding zero mating success	74	57	0.33	(0.10, 0.56)	0.012	0.47	(0.01, 0.79)	0.35	(0.00, 0.78)	0.01	(0.00, 0.27)
Laboratory studies	52	31	0.41	(0.13, 0.65)	0.007	0.72	(0.11, 0.92)	0.01	(0.00, 0.51)	0.01	(0.00, 0.41)
Field studies	58	46	0.41	(0.18, 0.61)	0.007	0.01	(0.00, 0.63)	0.71	(0.04, 0.89)	0.01	(0.00, 0.44)
Low-polyandry species	22	15	0.25	(0.00, 0.48)	0.056	0.02	(0.00, 0.56)	0.01	(0.00, 0.83)	0.01	(0.00, 0.78)
High-polyandry species	88	61	0.41	(0.15, 0.67)	0.005	0.75	(0.27, 0.92)	0.01	(0.00, 0.41)	0.01	(0.00, 0.31)

Table H. Predictors of inter-specific variation in female Bateman gradients excluding data on humans. Methodological moderators include mating success method (copulatory *versus* genetic mating success), mating success range (including *versus* excluding zero mating success category), study type (field *versus* lab) and year of publication (continuous variable). Effect of mating system contrasts low-polyandry and high-polyandry species. Effect of polyandry estimates the relationship between the female Bateman gradient and the proportion of polyandrous females in the population. Model estimates (i.e., estimated difference between groups) are shown as posterior modes with 95% Highest Posterior Density (HPD) intervals obtained from phylogenetically controlled General Linear-Mixed Effects Models. The variance explained by the moderator variable is given as the marginal R^2 with 95% HPD intervals in parentheses.

Moderator	Estimate		P_{MCMC}	R^2	
Mating success method	0.28	(0.09, 0.46)	0.003	0.16	(0.10, 0.21)
Mating success range	0.12	(0.04, 0.20)	0.002	0.03	(0.02, 0.05)
Study type	0.05	(-0.13, 0.22)	0.563	0.01	(0.00, 0.01)
Year of publication	0.00	(-0.02, 0.01)	0.528	0.00	(0.00, 0.01)
Mating system	0.27	(0.10, 0.43)	0.002	0.10	(0.05, 0.14)
Polyandry	0.60	(0.25, 0.94)	0.001	0.09	(0.05, 0.15)
