# Supplementary material for: Sexual selection in females and the evolution of polyandry
Source: PLoS Biol. 2023 Jan 10;21(1):e3001916. doi: 10.1371/journal.pbio.3001916 (PMC9831318; doi:10.1371/journal.pbio.3001916)
Supplement: S2 Text — (PDF) [file pbio.3001916.s002.pdf]

Supplementary Information – S2 Text

Sexual selection in females and the evolution of polyandry

Salomé Fromonteil[1¶], Lucas Marie-Orleach[2,3¶], Lennart Winkler[4], Tim Janicke[1,4*]

[1] CEFE, Univ Montpellier, CNRS, EPHE, IRD, Montpellier, France.

[2] Natural History Museum, University of Oslo, Oslo, Norway.

[3] CNRS, Université de Rennes 1, ECOBIO (Écosystèmes, biodiversité, évolution) - UMR 6553, Rennes, France.

[4] Applied Zoology, TU Dresden, Dresden, Germany.

[¶] Both authors contributed equally to this work.

* tim.janicke@cefe.cnrs.fr

This Supplementary Information file includes: References of primary studies

List of primary studies.

References of all 82 published primary studies included in the meta-analysis.

1. Andrade MCB, Kasumovic MM. Terminal investment strategies and male mate choice: Extreme tests of Bateman. Integrative and Comparative Biology. 2005;45(5):838-47. doi: 10.1093/icb/45.5.838.
2. Anthes N, David P, Auld JR, Hoffer JN, Jarne P, Koene JM, et al. Bateman gradients in hermaphrodites: an extended approach to quantify sexual selection. Am Nat. 2010;176(3):249-63.
3. Aronsen T, Berglund A, Mobley KB, Ratikainen II, Rosenqvist G. Sex ratio and density affect sexual selection in a sex-role reversed fish. Evolution. 2013;67(11):3243-57. doi: 10.1111/evo.12201.
4. Balenger S, Johnson L, Masters B. Sexual selection in a socially monogamous bird: male color predicts paternity success in the mountain bluebird, Sialia currucoides. Behav Ecol Sociobiol. 2009;63(3):403-11. doi: 10.1007/s00265-008-0674-5.
5. Barreto FS, Avise JC. Quantitative measures of sexual selection reveal no evidence for sex-role reversal in a sea spider with prolonged paternal care. Proceedings of the Royal Society B-Biological Sciences. 2010;277(1696):2951-6. doi: 10.1098/rspb.2010.0311.
6. Becher SA, Magurran AE. Multiple mating and reproductive skew in Trinidadian guppies. Proceedings of the Royal Society B-Biological Sciences. 2004;271(1543):1009-14. doi: 10.1098/rspb.2004.2701.
7. Bergeron P, Montiglio PO, Reale D, Humphries MM, Garant D. Bateman gradients in a promiscuous mating system. Behav Ecol Sociobiol. 2012;66(8):1125-30. doi: 10.1007/s00265-012-1364-x.
8. Bjork A, Pitnick S. Intensity of sexual selection along the anisogamy-isogamy continuum. Nature. 2006;441(7094):742-5.
9. Bolopo D, Canestrari D, Martinez JG, Roldan M, Macias-Sanchez E, Vila M, et al. Flexible mating patterns in an obligate brood parasite. Ibis. 2017;159(1):103-12. doi: 10.1111/ibi.12429.
10. Borgerhoff Mulder M. Serial monogamy as polygyny or polyandry? Human Nature. 2009;20(2):130-50. doi: 10.1007/s12110-009-9060-x.
11. Borgerhoff Mulder M, Ross CT. Unpacking mating success and testing Bateman's principles in a human population. Proceedings of the Royal Society B-Biological Sciences. 2019;286(1908):10. doi: 10.1098/rspb.2019.1516.

12. Broquet T, Jaquiery J, Perrin N. Opportunity for sexual selection and effective population size in the lek-breeding European treefrog (Hyla arborea) Evolution. 2009;63(3):674-83. doi: 10.1111/j.1558-5646.2008.00586.x.

13. Burkli A, Jokela J. Increase in multiple paternity across the reproductive lifespan in a sperm-storing, hermaphroditic freshwater snail. Mol Ecol. 2017;26(19):5264-78. doi: 10.1111/mec.14200.

14. Byers BE, Mays HL, Stewart IRK, Westneat DF. Extrapair paternity increases variability in male reproductive success in the chestnut-slded warbler (Dendroica pensylvanica), a socially monogamous songbird. Auk. 2004;121(3):788-95. doi: 10.1642/0004-8038(2004)121[0788:Epivim]2.0.Co;2.

15. Cattelan S, Evans JP, Garcia-Gonzalez F, Morbiato E, Pilastro A. Dietary stress increases the total opportunity for sexual selection and modifies selection on condition-dependent traits. Ecol Lett. 2020;23(3):447-56. doi: 10.1111/ele.13443.

16. Collet J, Richardson DS, Worley K, Pizzari T. Sexual selection and the differential effect of polyandry. Proc Natl Acad Sci U S A. 2012;109(22):8641-5. doi: 10.1073/pnas.1200219109.

17. Courtiol A, Pettay JE, Jokela M, Rotkirch A, Lummaa V. Natural and sexual selection in a monogamous historical human population. Proc Natl Acad Sci U S A. 2012;109(21):8044-9. doi: 10.1073/pnas.1118174109.

18. Croshaw DA. Quantifying sexual selection: a comparison of competing indices with mating system data from a terrestrially breeding salamander. Biol J Linnean Soc. 2010;99(1):73-83.

19. Dekker ML, Hagmayer A, Leon-Kloosterziel KM, Furness AI, Pollux BJA. High degree of multiple paternity and reproductive skew in the highly fecund live-bearing fish Poecilia gillii (Family Poeciliidae). Front Ecol Evol. 2020;8:14. doi: 10.3389/fevo.2020.579105.

20. Devost E, Turgeon J. The combined effects of pre- and post-copulatory processes are masking sexual conflict over mating rate in Gerris buenoi. Journal of Evolutionary Biology. 2016;29(1):167-77. doi: 10.1111/jeb.12772.

21. Fitze PS, Le Galliard JF. Inconsistency between different measures of sexual selection. Am Nat. 2011;178(2):256-68. doi: 10.1086/660826.

22. Fritzsche K, Arnqvist G. Homage to Bateman: sex roles predict sex differences in sexual selection. Evolution. 2013;67(7):1926-36. Epub 2013/07/03. doi: 10.1111/evo.12086.

23. Fuxjager L, Wanzenbock S, Ringler E, Wegner KM, Ahnelt H, Shama LNS. Within-generation and transgenerational plasticity of mate choice in oceanic

stickleback under climate change. Philos Trans R Soc B-Biol Sci. 2019;374(1768):12. doi: 10.1098/rstb.2018.0183.

24. Gagnon M-C, Duchesne P, Turgeon J. Sexual conflict in Gerris gillettei (Insecta: Hemiptera): influence of effective mating rate and morphology on reproductive success. Canadian Journal of Zoology. 2012;90(11):1297-306. doi: 10.1139/z2012-098.
25. Gao K, van Wijk M, Clement Z, Egas M, Groot AT. A life-history perspective on sexual selection in a polygamous species. Bmc Evolutionary Biology. 2020;20(1). doi: 10.1186/s12862-020-01618-3.
26. Garcia-Navas V, Ferrer ES, Bueno-Enciso J, Barrientos R, Sanz JJ, Ortego J. Extrapair paternity in Mediterranean blue tits: socioecological factors and the opportunity for sexual selection. Behavioral Ecology. 2014;25(1):228-38. doi: 10.1093/beheco/art111.
27. Gauthey Z, Tentelier C, Lepais O, Elosegi A, Royer L, Glise S, et al. With our powers combined: integrating behavioral and genetic data to estimate mating success and sexual selection. Rethinking Ecology. 2017;2:1.
28. Gerlach NM, McGlothlin JW, Parker PG, Ketterson ED. Reinterpreting Bateman gradients: multiple mating and selection in both sexes of a songbird species. Behavioral Ecology. 2012;23(5):1078-88. doi: 10.1093/beheco/ars077.
29. Glaudas X, Rice SE, Clark RW, Alexander GJ. The intensity of sexual selection, body size and reproductive success in a mating system with male-male combat: is bigger better? Oikos. 2020;129(7):998-1011. doi: 10.1111/oik.07223.
30. Gopurenko D, Williams RN, DeWoody JA. Reproductive and mating success in the small-mouthed salamander (Ambystoma texanum) estimated via microsatellite parentage analysis. Evolutionary Biology. 2007;34(3-4):130-9. doi: 10.1007/s11692-007-9009-0.
31. Gopurenko D, Williams RN, McCormick CR, DeWoody JA. Insights into the mating habits of the tiger salamander (Ambystoma tigrinum tigrinum) as revealed by genetic parentage analyses. Mol Ecol. 2006;15(7):1917-28. doi: 10.1111/j.1365-294X.2006.02904.x.
32. Greenway EV, Hamel JA, Miller CW. Exploring the effects of extreme polyandry on estimates of sexual selection and reproductive success. Behavioral Ecology. 2021;32(6):1055-63. doi: 10.1093/beheco/arab081.
33. Grunst AS, Grunst ML, Korody ML, Forrette LM, Gonser RA, Tuttle EM. Extrapair mating and the strength of sexual selection: insights from a polymorphic species. Behavioral Ecology. 2019;30(2):278-90. doi: 10.1093/beheco/ary160.

34. Hargrove JS, McCane J, Roth CJ, High B, Campbell MR. Mating systems and predictors of relative reproductive success in a Cutthroat Trout subspecies of conservation concern. Ecol Evol. 2021;11(16):11295-309. doi: 10.1002/ece3.7914.

35. Hoffer JNA, Marien J, Ellers J, Koene JM. Sexual selection gradients change over time in a simultaneous hermaphrodite. eLife. 2017;6:16. doi: 10.7554/eLife.25139.

36. Janicke T, David P, Chapuis E. Environment-dependent sexual selection: Bateman's parameters under varying levels of food availability. Am Nat. 2015;185(6):756-68.

37. Johannesson K, Saltin SH, Charrier G, Ring AK, Kvarnemo C, Andre C, et al. Non-random paternity of offspring in a highly promiscuous marine snail suggests postcopulatory sexual selection. Behav Ecol Sociobiol. 2016;70(8):1357-66. doi: 10.1007/s00265-016-2143-x.

38. Jokela M, Rotkirch A, Rickard IJ, Pettay J, Lummaa V. Serial monogamy increases reproductive success in men but not in women. Behavioral Ecology. 2010;21(5):906-12. doi: 10.1093/beheco/arq078.

39. Jones AG, Arguello JR, Arnold SJ. Validation of Bateman's principles: a genetic study of sexual selection and mating patterns in the rough-skinned newt. Proc R Soc Lond Ser B-Biol Sci. 2002;269(1509):2533-9.

40. Jones AG, Arguello JR, Arnold SJ. Molecular parentage analysis in experimental newt populations: The response of mating system measures to variation in the operational sex ratio. Am Nat. 2004;164(4):444-56. doi: 10.1086/423826.

41. Jones AG, Rosenqvist G, Berglund A, Arnold SJ, Avise JC. The Bateman gradient and the cause of sexual selection in a sex-role-reversed pipefish. Proc R Soc Lond Ser B-Biol Sci. 2000;267(1444):677-80.

42. Jones PH, Van Zant JL, Dobson FS. Variation in reproductive success of male and female Columbian ground squirrels (Urocitellus columbianus). Can J Zool-Rev Can Zool. 2012;90(6):736-43. doi: 10.1139/z2012-042.

43. Ketterson ED, Parker PG, Raouf SA, Nolan Jr V, Ziegenfus C, Chandler CH. The relative impact of extra-pair fertilizations on variation in male and female reproductive success in dark-eyed juncos (Junco hyemaus). In: Parker PG, Burley NT, editors. Avian Reproductive Tactics: Female and Male Perspectives. 491997. p. 81-101.

44. Krakauer AH. Sexual selection and the genetic mating system of Wild Turkeys. Condor. 2008;110(1):1-12. doi: 10.1525/cond.2008.110.1.1.

45. Kretzschmar P, Auld H, Boag P, Ganslosser U, Scott C, de Groot PJV, et al. Mate choice, reproductive success and inbreeding in white rhinoceros: New insights for conservation management. Evol Appl. 2020;13(4):699-714. doi: 10.1111/eva.12894.

46. LaBrecque JR, Alva-Campbell YR, Archambeault S, Crow KD. Multiple paternity is a shared reproductive strategy in the live-bearing surfperches (Embiotocidae) that may be associated with female fitness. Ecol Evol. 2014;4(12):2316-29. doi: 10.1002/ece3.1071.

47. Levine BA, Schuett GW, Clark RW, Repp RA, Herrmann HW, Booth W. No evidence of male-biased sexual selection in a snake with conventional Darwinian sex roles. R Soc Open Sci. 2020;7(10):10. doi: 10.1098/rsos.201261.

48. Levine BA, Smith CF, Schuett GW, Douglas MR, Davis MA, Douglas ME. Bateman-Trivers in the 21st Century: sexual selection in a North American pitviper. Biol J Linnean Soc. 2015;114(2):436-45. doi: 10.1111/bij.12434.

49. Levitan DR. Gamete traits influence the variance in reproductive success, the intensity of sexual selection, and the outcome of sexual conflict among congeneric sea urchins. Evolution. 2008;62(6):1305-16. doi: 10.1111/j.1558-5646.2008.00378.x.

50. Louder MIM, Hauber ME, Louder ANA, Hoover JP, Schelsky WM. Greater opportunities for sexual selection in male than in female obligate brood parasitic birds. Journal of Evolutionary Biology. 2019;32(11):1310-5. doi: 10.1111/jeb.13537.

51. Mangold A, Trenkwalder K, Ringler M, Hoedl W, Ringler E. Low reproductive skew despite high male-biased operational sex ratio in a glass frog with paternal care. Bmc Evolutionary Biology. 2015;15. doi: 10.1186/s12862-015-0469-z.

52. Marie-Orleach L, Janicke T, Vizoso DB, David P, Scharer L. Quantifying episodes of sexual selection: Insights from a transparent worm with fluorescent sperm. Evolution. 2016;70(2):314-28. doi: 10.1111/evo.12861.

53. McCullough EL, Buzatto BA, Simmons LW. Population density mediates the interaction between pre- and postmating sexual selection. Evolution. 2018;72(4):893-905. doi: 10.1111/evo.13455.

54. Mills SC, Grapputo A, Koskela E, Mappes T. Quantitative measure of sexual selection with respect to the operational sex ratio: a comparison of selection indices. Proceedings of the Royal Society B-Biological Sciences. 2007;274(1606):143-50. doi: 10.1098/rspb.2006.3639.

55. Mobley KB, Jones AG. Overcoming statistical bias to estimate genetic mating systems in open populations: a comparison of Bateman's principles between the

sexes in a sex-role-reversed pipefish. Evolution. 2013;67(3):646-60. doi: 10.1111/j.1558-5646.2012.01819.x.

56. Moorad JA, Promislow DEL, Smith KR, Wade MJ. Mating system change reduces the strength of sexual selection in an American frontier population of the 19th century. Evolution and Human Behavior. 2011;32(2):147-55. doi: 10.1016/j.evolhumbehav.2010.10.004.

57. Morimoto J, Pizzari T, Wigby S. Developmental environment effects on sexual selection in male and female Drosophila melanogaster. PLoS One. 2016;11(5):27. doi: 10.1371/journal.pone.0154468.

58. Munroe KE, Koprowski JL. Sociality, Bateman's gradients, and the polygynandrous genetic mating system of round-tailed ground squirrels (Xerospermophilus tereticaudus). Behav Ecol Sociobiol. 2011;65(9):1811-24. doi: 10.1007/s00265-011-1189-z.

59. Paczolt KA, Passow CN, Delclos PJ, Kindsvater HK, Jones AMG, Rosenthal GG. Multiple mating and reproductive skew in parental and introgressed females of the live-bearing fish Xiphophorus birchmanni. J Hered. 2015;106(1):57-66. doi: 10.1093/jhered/esu066.

60. Pelissie B, Jarne P, David P. Sexual selection without sexual dimorphism: Bateman gradients in a simultaneous hermaphrodite. Evolution. 2012;66(1):66-81. doi: 10.1111/j.1558-5646.2011.01442.x.

61. Poesel A, Gibbs HL, Nelson DA. Extrapair fertilizations and the potential for sexual selection in a socially monogamous songbird. Auk. 2011;128(4):770-6. doi: 10.1525/auk.2011.11127.

62. Pongratz N, Michiels NK. High multiple paternity and low last-male sperm precedence in a hermaphroditic planarian flatworm: consequences for reciprocity patterns. Mol Ecol. 2003;12(6):1425-33. doi: 10.1046/j.1365-294X.2003.01844.x.

63. Prosser MR, Weatherhead PJ, Gibbs HL, Brown GP. Genetic analysis of the mating system and opportunity for sexual selection in northern water snakes (Nerodia sipedon). Behavioral Ecology. 2002;13(6):800-7. doi: 10.1093/beheco/13.6.800.

64. Rios-Cardenas O. Patterns of parental investment and sexual selection in teleost fishes: Do they support Bateman's principles? Integrative and Comparative Biology. 2005;45(5):885-94. doi: 10.1093/icb/45.5.885.

65. Rodriguez-Munoz R, Bretman A, Slate J, Walling CA, Tregenza T. Natural and sexual selection in a wild insect population. Science. 2010;328(5983):1269-72.

66. Rose E, Paczolt KA, Jones AG. The contributions of premating and postmating selection episodes to total selection in sex-role-reversed Gulf Pipefish. Am Nat. 2013;182(3):410-20. doi: 10.1086/671233.

67. Saunders KM, Shuster SM. Bateman gradients and alternative mating strategies in a marine isopod. IntechOpen. 2019;(DOI: 10.5772/intechopen.88956). doi: DOI: 10.5772/intechopen.88956.

68. Scheepers MJ, Gouws G. Mating system, reproductive success, and sexual selection in Bluntnose Klipfishes (Clinus cottoides). J Hered. 2019;110(3):351-60. doi: 10.1093/jhered/esz008.

69. Schlicht E, Kempenaers B. Effects of social and extra-pair mating on sexual selection in blue tits (Cyanistes caeruleus) Evolution. 2013;67(5):1420-34. doi: 10.1111/evo.12073.

70. Schulte-Hostedde AI, Millar JS, Gibbs HL. Sexual selection and mating patterns in a mammal with female-biased sexual size dimorphism. Behavioral Ecology. 2004;15(2):351-6. doi: 10.1093/beheco/arh021.

71. Serbezov D, Bernatchez L, Olsen EM, Vollestad LA. Mating patterns and determinants of individual reproductive success in brown trout (Salmo trutta) revealed by parentage analysis of an entire stream living population. Mol Ecol. 2010;19(15):3193-205. doi: 10.1111/j.1365-294X.2010.04744.x.

72. Skjaervo GR, Roskaft E. Wealth and the opportunity for sexual selection in men and women. Behavioral Ecology. 2015;26(2):444-51. doi: 10.1093/beheco/aru213.

73. Tatarenkov A, Healey CIM, Grether GF, Avise JC. Pronounced reproductive skew in a natural population of green swordtails, Xiphophorus helleri. Mol Ecol. 2008;17(20):4522-34. doi: 10.1111/j.1365-294X.2008.03936.x.

74. Turnell BR, Shaw KL. High opportunity for postcopulatory sexual selection under field conditions. Evolution. 2015;69(8):2094-104. doi: 10.1111/evo.12721.

75. Ursprung E, Ringler M, Jehle R, Hodl W. Strong male/male competition allows for nonchoosy females: high levels of polygynandry in a territorial frog with paternal care. Mol Ecol. 2011;20(8):1759-71. doi: 10.1111/j.1365-294X.2011.05056.x.

76. Walker LK, Ewen JG, Brekke P, Kilner RM. Sexually selected dichromatism in the hihi Notiomystis cincta: multiple colours for multiple receivers. Journal of Evolutionary Biology. 2014;27(8):1522-35. doi: 10.1111/jeb.12417.

77. Wang X, Liu S, Yang YQ, Wu LN, Huang WH, Wu RX, et al. Genetic evidence for the mating system and reproductive success of black sea bream (Acanthopagrus schlegelii). Ecol Evol. 2020;10(10):4483-94. doi: 10.1002/ece3.6215.

78. Wells CP, Tomalty KM, Floyd CH, McElreath MB, May BP, Van Vuren DH. Determinants of multiple paternity in a fluctuating population of ground squirrels. Behav Ecol Sociobiol. 2017;71(2):13. doi: 10.1007/s00265-017-2270-z.

79. Whittingham LA, Dunn PO. Effects of extra-pair and within-pair reproductive success on the opportunity for selection in birds. Behavioral Ecology. 2005;16(1):138-44. doi: 10.1093/beheco/arh140.

80. Whittingham LA, Lifjeld JT. High paternal investment in unrelated young: extra-pair paternity and male parental care in house martins. Behav Ecol Sociobiol. 1995;37(2):103-8. doi: 10.1007/s0026550370103.

81. Williams RN, DeWoody JA. Reproductive success and sexual selection in wild eastern tiger salamanders (Ambystoma t. tigrinum). Evolutionary Biology. 2009;36(2):201-13. doi: 10.1007/s11692-009-9058-7.

82. Woolfenden BE, Gibbs HL, Sealy SG. High opportunity for sexual selection in both sexes of an obligate brood parasitic bird, the brown-headed cowbird (Molothrus ater). Behav Ecol Sociobiol. 2002;52(5):417-25. doi: 10.1007/s00265-002-0529-4.
