# Supplementary material for: Sexual selection in females and the evolution of polyandry
Source: PLoS Biol. 2023 Jan 10;21(1):e3001916. doi: 10.1371/journal.pbio.3001916 (PMC9831318; doi:10.1371/journal.pbio.3001916)
Supplement: Attachment — Submitted filename: ResponseToReviewers_PLoS_Biology_R1.pdf [file pbio.3001916.s003.pdf]

Response to Reviewers
PBIOLOGY-D-21-03049R1

Dear Editorial Board of PLoS Biology,

Thank you very much for the evaluation of our manuscript. We are genuinely very grateful for all the efforts made and especially for the insightful and constructive review. We now revised the manuscript substantially aiming to take all criticisms and suggestions into account.

First and foremost, we toned down and discuss more carefully the implications of our first major finding, which is a positive global effect size of female Bateman gradients. As suggested by the reviewer and the academic editor, we now put more emphasis on our second main finding, which is the positive relationship between the female Bateman gradient and the level of polyandry across the sampled taxa. This included extensive rewriting of the introduction and discussion sections and led to a change of the title of the manuscript.

Despite these changes, we still believe that our meta-analytic support of a positive Bateman gradient is an interesting finding in the context of sexual selection in females and we would like to briefly explain why we still want to publish this outcome. We fully agree with the reviewer that the Bateman gradient is an incomplete measure for the strength of sexual selection as it comes with several limitations. Furthermore, we admit that in the previous version we have failed to clearly explain all limitations and we were not critical enough when interpreting our results. However, some of the major concerns raised by the reviewer have already been addressed in the previous version (though not explicitly stated) by presenting an additional analysis on a subset of data that is not subject to putative biases (see detailed response to the reviewer below). In the revised version, we clarify the scope for potential biases in our analysis and provide further additional analyses, which we think help to refute some of the concerns raised.
This is not to say that the Bateman gradient is a perfect measure to quantify the strength of sexual selection - we agree that it is not and we now acknowledge the various drawbacks throughout the revised version - but we still believe that it provides an informative proxy for some components of sexual selection for the reasons outlined in Box 1 of the manuscript. We also would like to add that even if we did not preregister this study, it has been designed from the very beginning to test for the overall potential of sexual selection to operate in females and its role for the evolution of polyandry. Therefore, we feel that a complete change of the angle of the manuscript (removal of the sexual selection aspect and sole focus on the relationship with polyandry) only for the sake of publishing in a prestigious journal like PLoS Biology, would contribute to further publication bias on this subject – a problem that we are actually discussing in this manuscript.

To summarize, our finding of a positive female Bateman gradient across animals might appear somewhat surprising but it is based on a (unbiased) systematic literature search and therefore, in our view, representative for our current knowledge. Whether, a positive Bateman gradient is a powerful proxy for the actual strength of selection is debatable and we tried to be more transparent and critical when presenting its limitations and more

careful when drawing conclusions in the revised version of the manuscript following the reviewer's suggestions.

--

More specifically, the major changes in the revised version include:

1) We did a complete overhaul of the introduction and the discussion sections taking the specific reviewer's comments into account (see below). We toned down previous conclusions drawn from the positive global effect size of the female Bateman gradient taking the limitations of this metric into account and to prevent the impression that we are overselling our results.
2) We provide a more detailed introduction of the strengths and limitations of the Bateman gradient as a proxy for the strength of sexual selection by adding Box 1 "What Bateman gradients tell about sexual selection and what they do not" to the manuscript (supposed to appear as a one-page box in a printed version).
3) In the light of the outlined limitations, we now interpret the finding of a positive Bateman gradient not anymore as * clear-cut evidence* but as a *potential* for sexual selection to operate in females.
4) We updated the database by adding more recently published estimates of the female Bateman gradients so that the final sample size is now 120 effect sizes (before 111 effect sizes) from 84 primary studies. Inclusion of the 9 additional effect sizes did not change the model results qualitatively suggesting that the overall outcome of the statistical analysis is robust.
5) We added an additional analysis excluding human data as they may represent a special case in terms of how mating success has been estimated (mainly through the number of marriages).
6) We added an additional analysis in which we exclude sex-role reversed species to anticipate potential readers concerns wondering whether those systems are overrepresented in our database. This is because empirical tests of Bateman's principles in sex-role reversed species have often been considered as proof of concept. Interestingly, even when excluding sex-role reversed species from the dataset we obtain qualitatively identical results.

RESPONSES TO REVIEWER 1:

We are very grateful for all the detailed comments and constructive suggestions on the previous version. Moreover, many thanks for your willingness to discuss certain points with us afterwards.

--
R1.1: Some background first: There is a recent trend where the scientific community shouts that the work of 'old masters' wasn't nuanced enough. This is probably true, but I also think it is unhelpful to fall into the same trap from the opposite side, so to speak. The authors here really, really want to give an impression that sexual selection is the norm in females [as well as in males, which are not the focus here]. When reading the entire MS, they then

actually discuss caveats of this statement as well, but choose to ignore these caveats when summarizing their results in the abstract (lines 27-27) as well as in their introduction (e.g. line 77) and discussion (lines 166-168, and 284-287).

Response: In the revised version, we tried to be more careful when interpreting our results, always taking into account the limitations of our chosen proxy for the strength of sexual selection. Most importantly, we would like to stress that we have never intended to discredit the work of the 'old masters'. In fact, we are often surprised and in awe how comprehensive and prescient Darwin's and Bateman's ideas are – and many pieces of our own work actually support their claims/hypotheses. In the revised version, we tried our best to prevent giving the impression that the pioneers were fundamentally wrong or that we claim any "moral superiority". Both is certainly not true. This includes the clarification that it was actually Darwin himself who was the first to argue that sexual selection can operate in females (L 61 - 71).

--

R1.2: What are the caveats? There's an important conceptual one and also an important statistical one. Both are actually mentioned in the MS, but the authors in both cases seem not to really tackle them head-on, instead the reader is simply told that results should be robust. Let me go through both, before turning to smaller issues.

Major issue 1. The conceptual one is that a positive Bateman gradient is one of the two ingredients that ought to be in place for there to be sexual selection (since I decided to sign my review, let's also admit that I've emphasized this 'two requirements' aspect before, in ref.43 as it is cited here). The other, equally crucial one is that access to matings should be limiting. The authors seem to know this (they at least once remember to say that we're measuring a prerequisite, line 99, and elaborate on this problem on lines 175-187), but this insight doesn't permeate the MS as a whole. Everywhere else they instead follow their main track which is to state that these results suggest sexual selection to be the norm.
I've read this MS several times now and I am really not so sure the caveat is a minor one. Consider, for example, one of the species that is part of their dataset: the guppy. In one of their classic papers on this species, Magurran & Seghers 1994 estimated that a guppy female will experience a copulation attempt about once a minute. It is simply very, very hard for me to think of guppy females as sexually selected; it may well be that the gradient is positive (hard to know based on the supplementary material of this MS by the way, as data points aren't easy to match with the species), but the other part of the conceptual equation simply is not fulfilled. Guppy females are in fact estimated to suffer a lot from suboptimally high interest in them by males, to the extent that sexual harassment drives them to habitats they would otherwise not prefer (Darden & Croft 2008).

Response: We agree that the Bateman gradient captures only one prerequisite for sexual selection to operate. Access to mates also needs to be a limiting factor for competition to arise. As mentioned in the previous version, this is clearly a limitation of using Bateman gradients as a proxy for the strength of sexual selection. In the revised version we better acknowledge and provide more details regarding the relevance of this shortcoming in both the introduction (L 144-149; Box 1) and also in the discussion (L 303-331).

Nevertheless, we believe that there is one indication that females are potentially mate-limited in all of the included primary studies. Specifically, Bateman gradients can only be quantified if there is variance in mating success, which is, of course, true for any predictor variable in a linear regression. Importantly, variance in mating success implies that not all individuals obtain an optimal mating success (assuming that there is any). If we consider a species in which the female Bateman gradient is positive but females are not mate-limited (i.e. the case in which the Bateman gradient does not predict sexual selection), we would expect that all females achieve an optimal mating success, which eventually would lead to a scenario in which there should be no variance in mating success. This scenario is not the case for any of the included primary studies because mating success always varied in the tested populations so that the authors could actually examine the female Bateman gradient. Admittedly, variance in mating success may not only indicate mate limitation but may also arise from stochasticity and/or individual differences in optimal mating rates (however, if true, we would not expect to observe a non-zero Bateman gradient). For these reasons, it is probably an oversimplification to argue that variance in mating success equals mate-limitation but it represents at least a sign that there is a potential for mate limitation in females. In the revised version, we elaborate on this argumentation (Box 1; L 315-331). Again, this is not to say that Bateman gradients are robust with respect to the 2nd prerequisite - mate limitation - for sexual selection to operate. This is surely a limitation of our study and we make this very clear in the revised version. However, there is at least some indication that females might actually be mate limited in all primary studies.

Many thanks for picking up the guppy example. We have to admit, that we are not very familiar with this system, so that all arguments below are based on a somewhat superficial screen of the enormous body of literature on sexual selection in guppies. Yet to us, guppies actually may provide a wonderful showcase example for what we consider the merit of our study, i.e. to show that sexual selection in females is potentially (!) more widespread than we often think.
The primary studies included in our meta-analysis indicate that female Bateman gradients are positive in guppies (Becher & Magurran 2004) and therefore suggest that females 1) benefit from additional matings and 2) show variance in mating success so that there is scope for selection on mating success (we note that all data already have been made available with the previous submission so that readers can easily check the direction and significance of Bateman gradients in case this is too difficult to infer from the forest plot; for a table of all effect sizes please see https://salomefromonteil.github.io/META_SexSelFem/META_SexSelFem.html). Given the cited studies by the reviewer that demonstrate high mating rates and the drastic female response to male harassment, we agree that at the first glimpse it might be difficult to imagine that sexual selection can actually operate in female guppies. However, do these lines of evidence really imply that females are not under sexual selection and that positive Bateman gradients reported for this species are just statistical artefacts? For the following reasons, we believe that this conclusion might be somewhat premature. First, there is ample experimental evidence that female guppies benefit from multiple mating, demonstrating that there is a causal relationship between mating success and reproductive success in female guppies (e.g. (Evans & Magurran 2000)). This evidence is reassuring also because the primary studies on guppies that we included in our meta-analysis used genetic mating success, which is prone to lead to spurious estimates of the Bateman gradient (see response

to R1.3 below). Thus, in the case of guppies, the extracted positive Bateman gradient is likely to reflect an actual causal relationship even if the applied design is likely to lead to biased estimates. Yet, we totally agree, a causal relationship between mating success and reproductive success is only one prerequisite for sexual selection to operate. The second argument, why female guppies are potentially subject to sexual selection is that there seems to be solid evidence for male choice particularly with respect to body size. Several experiments suggest that males have a preference to copulate with larger (and therefore more fecund males) (Dosen & Montgomerie 2004; Herdman, Kelly & Godin 2004; Auld, Ramnarine & Godin 2017). If we define sexual selection in terms of selection arising from competition for mates and/or their gametes, we may need to establish a link between choice and competition – an issue that is probable debatable. We believe that mate choice imposes competition on the chosen sex because it makes them competing for being chosen. Importantly, competition does not necessarily require contest competition but can also occur indirectly in terms of scramble competition and also in terms of choice – all forms just impose a filter that leads to nonrandom success in mate acquisition. Coming now back to the guppy example, evidence of male choice for bigger females implies that females may compete for being chosen. In this case the sexually selected trait is not any kind of weaponry (as selected by contest competition) but body size. Body size is presumably also a naturally selected trait in females (bigger individuals have more resources and are therefore more fecund). However, given the evidence for male choice, the selection differential on body size is at least partly mediated by selection on mating success, i.e. the Bateman gradient (better illustrations of path analysis and decomposition of selection differentials into mating differentials and Bateman gradients can be found in (Anthes *et al.* 2017) and (Henshaw, Jennions & Kruuk 2018)). To summarize the second argument, sexual selection may operate on female guppies as there is evidence for male choice with body size being at least partially as sexually selected. By definition, the Bateman gradient quantifies the upper limit of sexual selection on body size.

Third, another line of evidence for sexual selection in female guppies comes from mating experiments in which female aggression has been quantified as a function of the adult sex ratio (ASR). Chuard, Brown and Grant (2016) conducted lab experiments in which they quantified signatures of intrasexual competition in both sexes testing a series of hypotheses. Interestingly, already in the abstract they argue that the historic “focus on describing the patterns of competition in males may have obscured the subtler and less frequent patterns of competition among females” – just in line with the ‘mantra’ of our manuscript. Their mating experiments suggest that aggression for access to mates depends on the ASR in both sexes. More male-biased ASR leads to more aggression among males and more female-biased ASR leads to more aggression among females. Maybe most importantly, they did not detect any sex differences in the rate of aggression. They conclude “Our most surprising result, however, was related to sex roles in the guppy: males were not more aggressive than females when competing for mates; if anything, the with-and-without- mate experiment indicated the reverse at an extremely high ASR.”. Under certain conditions, females even tended to be more aggressive than males. In a very recent paper, the same authors report a replication of their earlier study under seminatural conditions (Chuard, Grant & Brown 2022). They found that the ASR in the wild tended to be female-biased, which may contribute to competition among females for males in natural settings. Overall, they could largely replicate their earlier findings: female aggression increases with more female biased ASR. Moreover, they “found no evidence of higher intrasexual aggression rates in males

compared with females, contrary to our sex role predictions but similar to Chuard et al. (2016)." They argue that "In the wild, females are likely competing for males by being aggressive towards other females, potentially until subordinate females leave the pool." The most crucial question is, of course, whether aggression among females reflects competition for mates or whether they actually compete for food. In both contributions, the authors provide some arguments against the competition-for-food hypothesis (e.g. under lab conditions food was provided ad libitum) and refer to another studies suggesting that " both sexes seem to value these fitness-related resources equally (Chuard, Brown & Grant 2018). Still, the alternative hypothesis of competition for food among females can definitely not ruled out.

Taken together, evidence for sexual selection in female guppies is clearly limited and we don't claim to be experts on sexual selection in these fish but there are at least some indications that it may play a role in females even though less than in males. We can imagine that our knowledge on and the quest for sexual selection in female guppies is exemplary for many taxa and therefore illustrates one of the main messages of our study: Until now, the vast majority of sexual selection research on guppies has been focused on males (i.e. male-male competition, female choice). This may correspond to true sex differences because sexual selection is stronger on males leading to more elaborated male sexually selected traits (e.g. male coloration in guppies). However, does this necessarily imply that sexual selection is absent in females? Given the above-outlined empirical evidence, we would argue that it is far too early to draw this conclusion. The well-founded theoretical support for Darwinian sex roles to predominate sexually reproducing organisms and the massive publication bias towards empirical studies on sexual selection on males, may make us sometimes believe that sexual selection in females is rare just because 1) it is weaker than compared to males, 2) it is more difficult to detect (due to the general lack of conspicuous female ornaments and armaments; (Berglund 2013)) and 3) because sometimes we may unintentionally consider absence of evidence as evidence of absence. Neither the above-cited studies on guppies nor our meta-analysis can provide an ultimate proof for sexual selection in females, we agree on that, but we believe that both studies are meaningful in the sense that they highlight a potential for sexual selection to be more widespread in females than often assumed – either within certain species (such as studies on male choice and female-female aggression suggest in guppies) or across species (such as our meta-analysis on Bateman gradients).

In the revised version of our manuscript, we tried to be more careful when interpreting our results. For instance, we removed the claim that our findings suggest that sexual selection in females is the norm. Instead, we better acknowledge the limitations of our study and clearly state that our meta-analytic evidence suggests the 'potential' for sexual selection in females to be more widespread than often assumed. Thereby, we hope that one of our two major findings (a positive Bateman gradient across species) may stimulate more empirical research testing for actual female-female competition in females (also in species with Darwinian sex roles) and also encourage more theoretical work on questions such as 'Does sexual selection on females mainly select for traits that are also under natural selection (such as body size in guppies) but not for conspicuous ornaments and armaments as in males?' (see also (Berglund 2013)).

--

R1.3: The fact that the authors relegate the caveat-discussion to a role where it needs to be stated (lines 175-187), but only presented as just one 'alternative' (line 182) when it actually is a core problem in this type of analysis, is also evident in the introduction. Here, twice (lines 56-57, and 64-65), the authors assert a causality where benefits of multiple mating leads to (without caveats) selection for competing.

Response: As stated above, we revised the introduction and discussion sections extensively acknowledging limitations of Bateman gradients (see Box 1, introduction: 141-149, discussion: 308-331, 342-361), toning down our conclusions and avoiding inference of causality based on our results. However, we believe that positive selection on mating success (i.e. a positive Bateman gradient) is still a cause (though not the only one) for precopulatory sexual selection (just as argued by Bateman himself). Mate limitation is not enough for sexual selection to arise, there must also be a fitness benefit for outcompeting rivals. Again, none of our data allow inference of causality, and we acknowledge this in the manuscript (Box 1, L 334-337, L346-352) but conceptually we believe it is valid to argue that there is a causal relationship between selection on mating success and competition for mates.

--

R1.4: Major issue 2. The statistical problem, also somewhat alluded to here but not really tackled, is that clearly identified by Gerlach et al. 2012 (which is cited in the supplementary as a data source, but its key message is not really appreciated by the current authors). They point out that a positive Bateman gradient need not imply selection, for the causality can be different: it is easier to find genetic evidence for multiple mating in large clutches, thus if a female is more fecund (for whatever reason), one may be able to find it to have mated multiply more easily - statistically speaking. This made Gerlach et al. write, very sensibly, that "Both sexes exhibited a strong positive Bateman gradient, even when the number of breeding years was accounted for. Although theory suggests that this pattern indicates a strong potential for sexual selection in both sexes, we argue that the interpretation of strong Bateman gradients, particularly in females, has many potential complications [...] Because neither of these explanations requires that increased mating success causes increased reproductive success, we conclude that using Bateman gradients to measure the potential for sexual selection may be misleading for some mating systems and life histories, such as the iteroparous social monogamy found in juncos." The current MS's authors cite related literature, but do not really adhere to this message, and appear to give the impression that the relevant causality is related to male mate choice for fecund females (which of course can also exist, but note that the above problem of causal inference exists even if no male is choosy).

Response: We appreciate this comment and agree that the problem discussed by Gerlach *et al.* (2012) may lead to spurious estimates of the Bateman gradient. However, this problem only arises if mating success is measured based on genetic parentage (i.e. the number of mates a focal individuals has genotyped offspring in common) as already discussed earlier by Anthes *et al.* (2010) and later in Anthes *et al.* (2017), Marie-Orleach *et al.* (2016) and Collet *et al.* (2014). We admit that in the previous version, we failed to make this point clear enough by mentioning it only in the methods section “Quantifying mating success in terms

of the number of genetic parents may not only obscure a potentially important component of post-copulatory sexual selection (because unsuccessful copulations and multiple copulations with the same partner remain undetected) but also leads to an autocorrelation of mating success and reproductive success, particularly in species with low fecundity". However, our awareness of this problem was the underlying motivation to test for an effect of the mating success method on estimates of the Bateman gradient and whether studies relying on copulatory mating success still show evidence for positive female Bateman gradients.

In the revised version, we mention this issue already in the introduction and in the discussion sections (Box 1, L 366-375) to avoid the impression that we do not acknowledge this limitation and clarify that we actually can address this statistical problem to the extent that even the analysis of a subset of studies using copulatory mating success (i.e., those studies that are definitely not subject to this bias) still provide evidence for positive Bateman gradients in females.

Moreover, we provide an additional analysis testing for a negative relationship between female fecundity and Bateman gradients in studies using genetic mating success. Such a relationship is expected if Bateman gradients are driven by the stressed statistical problem because it primarily arises if fecundity of a given species is low so that every increase in genetic mating success must also imply an increase in reproductive success such as Juncos and other birds. Reassuringly, we did not find such a statistically significant negative relationship, suggesting that positive Bateman gradients in studies using genetic mating success cannot only be explained by the intrinsic dependence of reproductive success and mating success (L 511-520). This also corresponds to the above-discussed example of guppies: The included primary studies used genetic mating success and report positive female Bateman gradients. As mentioned before, there are several experimental studies on guppies in which mating success was manipulated that support a causal relationship between multiple mating and female reproductive success.

We also note that to us the intrinsic relationship between genetic mating success and reproductive success is not a problem of causality but in fact an autocorrelation (see also Anthes *et al.* (2010), Anthes *et al.* (2017) and Arnqvist (2013).

In our opinion, the major shortcoming of the Bateman gradient is - as is true for all selection gradients - it does not imply causality unrelated to the autocorrelation in studies using genetic mating success. As discussed in the previous version of the manuscript, the female Bateman gradient can be positive because females with higher fecundity (or any other fitness related trait) might be more attractive mating partners, which reverses the underlying causality of Bateman gradients. We highlight this limitation in more detail in the revised version (Box 1, L 334-337, L 346-352). Reassuringly, at least in some species such as guppies for which there is evidence that males have indeed a preference for larger females, there is experimental evidence for a causal relationship between mating success and reproductive success.

--

Minor (or moderately important) issues:

R1.5: minor issue 1. (a) I was confused by the term 'monandry' the way it is used in the MS. Monandry means, literally, that a female has only 1 mate, so how come one can estimate a

Bateman gradient for someone who never has more than 1? By looking at the species list, it appears that 'monandry' is used for species such as tree frogs, humans, cowbirds & bluebirds, ground squirrels and the like. I know birds better than these other species; at least extrapolating from birds, I think you mean something like social (but not genetic) monogamy here. I'm not sure how this would apply to e.g. frogs, but the terminology used in the classification needs revisiting in any case.

Response: Agreed. In the previous version we tried to clarify that "species classified as monandrous are not strictly monandrous in the sense of having only one mating partner (for which Bateman gradients cannot be estimated because there is no variation in mating success). Instead, monandrous species also mate multiply but at a lower frequency compared to polyandrous species.". However, we see that the used terminology might still be misleading and addressed this issue by using the terms "low-polyandry" and "high-polyandry" throughout the revised version. Many thanks for raising this point!

--

R1.6: (b) As an aside, it is a little bit of a shame that the results are not subdivided at all into taxon-specific statements; the biology of multiple mating can be quite different across taxa, and even if statistical significance was lost, it'd be informative to present data colour-coded for taxon, or something like that.

Response: Thanks. In the revised version we report effect sizes for all major classes covered in our meta-analysis in the supplementary material (Table S2), suggesting that fish show stronger female Bateman gradients compared to all other taxa probably because of the prominent presence of sex-role reversed species (pipefish and seahorses).

--

R1.7: minor issue 2. I was quite surprised by the statement on lines 246-248, that Shuker & Kvarnemo claim direct benefits provided by mates to be a phenomenon that falls under natural selection only, and that only indirect selection for complementary or good genes 'qualifies' (in a 'sexual selection due to mate choice' context). I was one of the reviewers of S&K and surely would have questioned their logic there had they promoted such an extreme view. I reread S&K now again, including explicitly checking every occurrence of the words 'direct' and 'indirect', 'good genes' or 'complementary genes'. Direct fitness effects are talked about a lot by them, but not in the way the authors of the current MS claim; the contrast to indirect selection based on good genes or complementary genes is definitely not made - the only place where the word 'indirect' is present in S&K is to create a contrast to indirect fitness in another field of study, and neither good nor complementary genes are discussed at all. Which in itself may be surprising, but so it is, and it would be good if the current authors did not misrepresent S&K's opinions.

Response: The reviewer kindly gave us the opportunity to discuss this issue in person and we seemed to have largely agreed on that there is a potential issue with the recently proposed definition of sexual selection by Shuker and Kvarnemo (2021). However, this clearly needs more detailed argumentation, which is probably out of the scope of the

current manuscript. Therefore, we excluded the discussion of Shuker & Kvarnemo's definition in the revised version of the manuscript and plan to publish it as a stand-alone paper on this topic.

--

R1.8: To come back to the main issue: how to make sure that the authors don't swing the pendulum to an equally untenable position as where the 'old masters' have arguably been the other way? I fear that if the authors boil the message down to statements of sexually selected females being the norm, they risk precisely that, for the two major issues above have simply not been dealt with here to an extent that they could be safely stated to be unimportant. Also - though this is perhaps more like a personal preference of mine, and I intentionally use words here that may sound too strong, but here goes - to me it is a little distasteful to work as if one wanted to 'cancel' people when their 'crime' is really only to have first tackled those phenomena within a field that are more obvious (showy males, and why females are much less often showy). Personally I wouldn't state 'more studies on the more obvious question' as a bias. One can definitely, as a field, move towards the more subtle and harder to study questions without having to express, between the lines, some sort of moral superiority about this progress. One real scientific question, actually, that arises in my own mind here: if it is true (think of the guppies again) that male availability may be abundant for females in many systems, yet one can find evidence of a positive Bateman gradient… why didn't all females then mate so much that they would have reached those mating rates that yield best fitness? I believe this is the real, nuanced question in a lot of these systems.
Also, I found a couple of messages in the MS much more robust, and insightful, than the one that is on more shaky ground & chosen as the main emphasis of the authors. Those neat messages are on lines 202-205 and 281-282. If I were to write a MS based on this sort of data, I'd go explanding those messages a lot more, and avoid any sensationalist claims that everything in the past has been just an unfortunate consequence of people being biased.

Response: As explained above, we tried to be more careful when interpreting our results taking the two major concerns into account. Most importantly to us, we tried to avoid any statements that might sound as if we discredit the contributions of the 'masters' of sexual selection research. This was and is not our intention. Moreover, as suggested by the reviewer, we put more emphasis on the second major outcome of our study which is the relationship between Bateman gradients and the level of polyandry.

Once again, many thanks for the very thoughtful and stimulating comments on the previous version.

REFERENCES

Anthes, N., David, P., Auld, J.R., Hoffer, J.N., Jarne, P., Koene, J.M., Kokko, H., Lorenzi, C.M., Pélissié, B., Sprenger, D., Staikou, A. & Schärer, L. (2010) Bateman gradients in hermaphrodites: an extended approach to quantify sexual selection. *American Naturalist,* **176,** 249-263.

Anthes, N., Häderer, I.K., Michiels, N.K. & Janicke, T. (2017) Measuring and interpreting sexual selection metrics – evaluation and guidelines. *Methods in Ecology and Evolution,* **8,** 918-931.
Arnqvist, G. (2013) Comment on "Bateman in nature: predation on offspring reduces the potential for sexual selection". *Science,* **340,** 2.
Auld, H.L., Ramnarine, I.W. & Godin, J.G.J. (2017) Male mate choice in the Trinidadian guppy is influenced by the phenotype of audience sexual rivals. *Behavioral Ecology,* **28,** 362-372.
Becher, S.A. & Magurran, A.E. (2004) Multiple mating and reproductive skew in Trinidadian guppies. *Proceedings of the Royal Society B-Biological Sciences,* **271,** 1009-1014.
Berglund, A. (2013) Why are sexually selected weapons almost absent in females? *Current Zoology,* **59,** 564-568.
Chuard, P.J.C., Brown, G.E. & Grant, J.W.A. (2016) The effects of adult sex ratio on mating competition in male and female guppies (Poecilia reticulata) in two wild populations. *Behavioural Processes,* **129,** 1-10.
Chuard, P.J.C., Brown, G.E. & Grant, J.W.A. (2018) Competition for food in 2 populations of a wild-caught fish. *Current Zoology,* **64,** 615-622.
Chuard, P.J.C., Grant, J.W.A. & Brown, G.E. (2022) Mating competition and adult sex ratio in wild Trinidadian guppies. *Behavioral Ecology,* **33,** 892-900.
Collet, J.M., Dean, R.F., Worley, K., Richardson, D.S. & Pizzari, T. (2014) The measure and significance of Bateman's principles. *Proceedings of the Royal Society B-Biological Sciences,* **281,** 9.
Dosen, L.D. & Montgomerie, R. (2004) Female size influences mate preferences of male guppies. *Ethology,* **110,** 245-255.
Evans, J.P. & Magurran, A.E. (2000) Multiple benefits of multiple mating in guppies. *Proceedings of the National Academy of Sciences of the United States of America,* **97,** 10074-10076.
Gerlach, N.M., McGlothlin, J.W., Parker, P.G. & Ketterson, E.D. (2012) Reinterpreting Bateman gradients: multiple mating and selection in both sexes of a songbird species. *Behavioral Ecology,* **23,** 1078-1088.
Henshaw, J.M., Jennions, M.D. & Kruuk, L.E.B. (2018) How to quantify (the response to) sexual selection on traits. *Evolution,* **72,** 1904-1917.
Herdman, E.J.E., Kelly, C.D. & Godin, J.G.J. (2004) Male mate choice in the guppy (Poecilia reticulata): Do males prefer larger females as mates? *Ethology,* **110,** 97-111.
Marie-Orleach, L., Janicke, T., Vizoso, D.B., David, P. & Schärer, L. (2016) Quantifying episodes of sexual selection: Insights from a transparent worm with fluorescent sperm. *Evolution,* **70,** 314-328.
Shuker, D.M. & Kvarnemo, C. (2021) The definition of sexual selection. *Behavioral Ecology*.
