# Supplementary material for: Sexual selection in females and the evolution of polyandry
Source: PLoS Biol. 2023 Jan 10;21(1):e3001916. doi: 10.1371/journal.pbio.3001916 (PMC9831318; doi:10.1371/journal.pbio.3001916)
Supplement: Attachment — Submitted filename: PLoS guppy musings.pdf [file pbio.3001916.s005.pdf]

First of all, apologies that I had to ask for extra time to re-review this paper, August has been a rather crammed month for me.

I (still) think that this is an important paper, and therefore it would be good to make sure that its results are not misinterpreted by the wider audience. I think there remains some discrepancy between the authors' views and those of mine, and I do realize that it may be too much to hope that they'd completely write it "my way". After all, it is much more tempting to make strong general claims – to borrow the authors' term from the response letter, to follow a 'mantra' – than to water a story completely down with all the caveats that exist. Right now, their solution is to mention caveats abundantly (which I appreciate, a lot) but then, simultaneously, provide summaries where the most exciting (caveat-free) interpretation is favoured. I understand this, even if it does make me tear my hair a little at places.

What I will do below is to reiterate some points, because the above 'have & eat the cake' approach makes me actually a little unsure how well the authors have really internalized the causality issue. Perhaps this review letter serves at least as some discussion of how thorny the issues are. I should also say that I fully see that the authors seem to understand what I'm saying below (as evidenced by e.g. line 222-223) but then I also seem to find plenty of evidence that they would, often, much rather look away from this problem (this is what I mean by having & eating the cake: now that we 'have' it, i.e. have discussed it, we can eat it, and focus on the phenomenon as if the caveats weren't there). I could pick many sentences in the response letter to justify me saying this, sometimes even in the same sentence: e.g., when they write 'some indication that females might be mate limited in all primary studies', the words 'some' and 'might' of course show that the authors remember adding caution is necessary, but then there is this word 'all' that makes the claim actually really strong: the authors do believe that it is possible that the caveats do not apply to **any** of the species, and that mate limitation really is the underlying cause of all these positive Bateman gradients.

A.

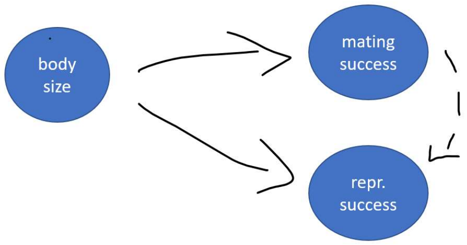

B.

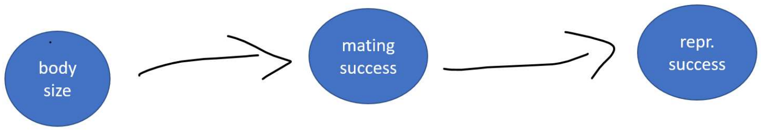

So, let's talk about guppies once more. Are female guppies more like (A) below or like (B) above? What I drew there are arrows that are causal (solid line), and arrows that are non-causal (dashed line). Both (A) and (B) predict a positive Bateman gradient. In (A) it arises because males target highly fecund, large females more often. It is even possible, let's call this scenario A2, that the dashed arrow becomes solid (causal) but **negative** (= many matings are bad for females), and **still** the Bateman gradient remains positive. How come? Consider the possibility that reproductive success (for any given body size) is lowered a little when the female mates a lot; if the fecundity advantage of a large female (based on the causal arrow from large bodies to many eggs) 'shines

through' strongly enough, the measured Bateman gradient can stay positive despite many matings being bad for her (as well as for any female of any size).

Scenario (B) is, out of the two, the only one where females are sexually selected: body size there is a sexually selected trait **because** females with small bodies fail to have enough male attention. The 2nd arrow is the one that states that there can be such a distinction between enough/not enough (i.e., mate limitation). Experimentally, one would have to show that the rightmost arrow in (B) really exists independently of the leftmost one, i.e., if an otherwise unattractive (= small-bodied) female is given one extra mate 'for free', her reproductive success increases. This is the mate limitation arrow.

To be a bit blunt, I am not aware of such a study in guppies. So, how to read the statement (in the response letter) of the authors of the current MS that for guppies, the "causality" has been experimentally proven? Which precise causality are we talking about? The papers cited by the authors (in the response letter) actually actively argue against (B)'s mate-limitation, and the arrows that are discussed tend to be much more aligned with (A), as we know for sure that male guppies prefer large females. Researchers actively state facts against (B), even those who the authors cited to prove their point. Chuard et al. 2016 clearly say that females can store sperm and thus do not even need a new mate to produce a new brood, and that therefore it's unlikely that they're fighting for access to males per se. In both of their papers (2016 and 2022) they discuss the option that females may be aggressively competing for food too. In 2016, they strongly favour the idea, which they consider much more plausible than mate limitation per se, that females may compete for access to *specific* males. Now, this is not a feature of either (A) or (B), but much more subtle, but it is a phenomenon that is hard to capture with Bateman gradients (I think I've written somewhere about how Bateman doesn't distinguish between mate identities at all, which is a limitation of its scope but not a flaw per se; but can't remember right now where I wrote that. And in any case, that is about the boundaries of definitions of sexual selection, i.e. the topic of the paper you are writing in the future, not this one…)

To come back on topic: the issue remains that the authors in their reply did not really engage with the literature I suggested they look at, where it is described how actively female guppies seem to avoid males. This is simply hard to reconcile with the notion 'the system is driven by more matings being good, and if someone failed to reach many matings, it's because of mate-limitation'. This is what we (as a field) are grappling with: there is definitely evidence for more subtle and complicated sexual interactions than any simplistic categorization would allow; this I wholeheartedly agree with with the authors; but this does not necessarily mean mate-limitation in both sexes, and all this has to be interpreted together with evidence such as – to mention a paper here that I did not mention before – Killen et al. 2016 Funct Ecol – that female guppies exposed to higher levels of male harassment spend so much time and energy 'exercising' that they develop more efficient swimming, improving their aerobic capacity in the process. Why spend all this effort swimming in avoidance contexts if they're mate-limited to begin with?

It might also be good to use scenario (A) to reconsider the authors' statement: "mating success may not only indicate mate limitation but may also arise from stochasticity and/or individual differences in optimal mating rates (**however, if true, we would not expect to observe a non-zero Bateman gradient**) [emphasis added]." If we add stochastic mating rates to my scenario (A), then the existing causal arrows can completely produce a non-zero Bateman gradient while the mating rates vary between females. It's just that the variation in reproductive success isn't explained by the additional matings that some females got; it's reflective of them happening to be the larger females who can produce more eggs.

OK, sorry, I've written way too much already. Basically, all these words I am producing here are about parsimony. If we wanted to keep the 2nd causal arrow in (B), expressable as "if only she had managed to mate one more time, she'd have higher reproductive success" valid, we would have to pay a pretty big parsimony cost when explaining all behaviours of guppies that become a bit weird under that assumption, such as the significant effort that females seem to put into avoidance of males (and matings). (As an aside, in many phrases in the MS, I would actually welcome a neutral term 'number of matings' rather than 'mating success', as the latter evokes a feeling of succeeding being difficult, i.e. it is an invitation to assume that mate-limitation exists in the system.)

OK, enough about guppies! I actually wish a real guppy biologist would have a look at this discussion. I'll make a few remarks on the MS text itself now, but each of them is simply reacting to the feeling of the authors wanting to avoid watering down the message and therefore having a bit of a 'have their cake and eat it too' thing in there. Ultimately it's up to them – or perhaps the editor – to decide how much they want to keep boldly claiming that all this really is evidence for sexual selection in the mate-limitation sense, when I'd say it's probably sometimes really there and sometimes really, really, really not.

Abstract: No caveats are mentioned here at all (could insert sad emoji here, knowing how many people read the abstract only)

Line 173 I would actually say that comparisons across contexts are the most dangerous ones of them all… I think I agree with this sentiment if an "all else being equal" meaning is meant, but across contexts (e.g. sexes), much is typically not equal.

183 it informs of this *if* it is not a result of an 'A' type situation

223 Here I agree wholeheartedly! See all of above for why I am sometimes confused: you seem to understand this issue so perfectly here… and prefer to not carry it through in other places.

267 This, to me, appears to indicate that the detection bias (identified by Gerlach) is a real issue (compare this with the response letter's comparison saying why it might not be). Who knows…

300 Now you definitely claim to have demonstrated "strength of selection for mate acquisition" to be the driver of patterns, the caveats are not remembered. Lines 303-304, ditto. Line 305 onwards, now you admit that the above claims actually may or may not be true. I know people should never take individual sentences out of context, but precisely this will happen when using this sort of flow in one's paragraphs.

315 This made me think of 'number of mates', 'mating rate' or similar neutral phrases, vs. 'mating success' where the latter phrase makes one automatically think there is a directional prediction and that failures are a real possibility. See my comment far above somewhere, in brackets.

329-330 Actually, there's not just these two options. It could also be that there is no mate limitation (for females), also no stochasticity, but a situation (A) as described above, with some females being larger than others and this leading to deterministically more mates for them. The point is that the small females might also have a totally sufficient number of mates, they're just less fecund because they're small (and extra mates won't make them more fecund because this doesn't change body sizes after all. Note that there are systems where matings yield a lot of resources, e.g. many insects, and then fecundity or even lifespan may actually causally increases with the number of matings!)

351 …OK, now you do mention this! But would be good to merge with what's there in 329-330? Mostly, I think my verbose comments are really about the 'separation' between main claims and

‘issues’, if they were better woven into a single coherent argument I’d be really happy about everything.

378-381 Here you make strong (though implicit) causality claims (you assume ”we can say B, because A has been somehow excluded”)

391 Would it actually be honest to mention the complications about one particular species, for illustration of how thorny this is after all? Given that the review process has made us all scratch our heads a lot about guppies, could some of the arguments presented in this interaction be openly discussed here, to provide an illustrative example?

OK, this is one of the longest reviews I’ve ever written. Apologies! Also, I hope I’m not giving the impression that I’m reluctant to accept any evidence for sexual selection in females. It’s much more about celebrating the diversity and trying to be parsimonious at the same time. Plus, perhaps I worry that meta-analyses, for all their benefits, tend to hope to uncover rules of a ‘one size fits all’ type, i.e. that something operates essentially similarly across taxa. I’m pretty sure there can be (A)-type as well as (B)-type situations out there, and probably (C) and (D) as well (the guppies after all might compete for a *subset* of current mating opportunities)… how should one then think about the distributions of effect sizes that one sees in pooled data? I may want to write something about this one day, but that day is not today!

(By the way, my scenario B could also be modified to have an extra arrow, a direct causal one from body size to reproductive success. I am aware of that but the above was simpler to write assuming the absence of that, including it wouldn’t really change the main points.)

Hanna Kokko (once again)
