# Supplementary material for: Sexual selection in females and the evolution of polyandry
Source: PLoS Biol. 2023 Jan 10;21(1):e3001916. doi: 10.1371/journal.pbio.3001916 (PMC9831318; doi:10.1371/journal.pbio.3001916)
Supplement: Attachment — Submitted filename: Fromonteil_et_al_PLoS_Biol_R2_ResponseToReviewers_v02.pdf [file pbio.3001916.s006.pdf]

Response to Reviewers
PBIOLOGY-D-22-01567R1

Dear Editorial Board of PLoS Biology,

Once again, thank you very much for handling our manuscript and for your positive feedback. We are very grateful for all the efforts made and especially for the stimulating review. We revised the manuscript aiming to take all criticism and suggestions into account. In addition, we also changed the statistical analysis for detecting publication bias following the recommendations made in a very recent article by Nakagawa *et al.* (2022).

RESPONSES TO REVIEWER 1:

Many thanks for these very interesting and thought-provoking reflections on the guppy system, which might indeed be exemplary for many other species included in our meta-analysis. As mentioned earlier and in the manuscript, we agree that the main shortcomings of the Bateman gradient are 1) the relationship between mating success and reproductive success may not be causal but correlational and 2) the second prerequisite for sexual selection to operate may not hold, i.e. that females have limited access to males. As detailed out below in the responses to the more specific comments, we made further efforts to highlight these limitations throughout the revised manuscripts and tried to avoid making too strong statements.

--
R1.1: So, let's talk about guppies once more. Are female guppies more like (A) below or like (B) above? What I drew there are arrows that are causal (solid line), and arrows that are non-causal (dashed line). Both (A) and (B) predict a positive Bateman gradient. In (A) it arises because males target highly fecund, large females more often. It is even possible, let's call this scenario A2, that the dashed arrow becomes solid (causal) but negative (= many matings are bad for females), and still the Bateman gradient remains positive. How come? Consider the possibility that reproductive success (for any given body size) is lowered a little when the female mates a lot; if the fecundity advantage of a large female (based on the causal arrow from large bodies to many eggs) 'shines through' strongly enough, the measured Bateman gradient can stay positive despite many matings being bad for her (as well as for any female of any size). Scenario (B) is, out of the two, the only one where females are sexually selected: body size there is a sexually selected trait because females with small bodies fail to have enough male attention. The 2nd arrow is the one that states that there can be such a distinction between enough/not enough (i.e., mate limitation). Experimentally, one would have to show that the rightmost arrow in (B) really exists independently of the leftmost one, i.e., if an otherwise unattractive (= small-bodied) female is given one extra mate 'for free', her reproductive success increases. This is the mate limitation arrow.
To be a bit blunt, I am not aware of such a study in guppies. So, how to read the statement (in the response letter) of the authors of the current MS that for guppies, the "causality" has been experimentally proven? Which precise causality are we talking about? The papers

cited by the authors (in the response letter) actually actively argue against (B)'s mate-limitation, and the arrows that are discussed tend to be much more aligned with (A), as we know for sure that male guppies prefer large females. Researchers actively state facts against (B), even those who the authors cited to prove their point. Chuard et al. 2016 clearly say that females can store sperm and thus do not even need a new mate to produce a new brood, and that therefore it's unlikely that they're fighting for access to males per se. In both of their papers (2016 and 2022) they discuss the option that females may be aggressively competing for food too. In 2016, they strongly favour the idea, which they consider much more plausible than mate limitation per se, that females may compete for access to specific males. Now, this is not a feature of either (A) or (B), but much more subtle, but it is a phenomenon that is hard to capture with Bateman gradients (I think I've written somewhere about how Bateman doesn't distinguish between mate identities at all, which is a limitation of its scope but not a flaw per se; but can't remember right now where I wrote that. And in any case, that is about the boundaries of definitions of sexual selection, i.e. the topic of the paper you are writing in the future, not this one…). To come back on topic: the issue remains that the authors in their reply did not really engage with the literature I suggested they look at, where it is described how actively female guppies seem to avoid males. This is simply hard to reconcile with the notion 'the system is driven by more matings being good, and if someone failed to reach many matings, it's because of mate-limitation'. This is what we (as a field) are grappling with: there is definitely evidence for more subtle and complicated sexual interactions than any simplistic categorization would allow; this I wholeheartedly agree with the authors; but this does not necessarily mean mate-limitation in both sexes, and all this has to be interpreted together with evidence such as – to mention a paper here that I did not mention before – Killen et al. 2016 Funct Ecol – that female guppies exposed to higher levels of male harassment spend so much time and energy 'exercising' that they develop more efficient swimming, improving their aerobic capacity in the process. Why spend all this effort swimming in avoidance contexts if they're mate-limited to begin with?

It might also be good to use scenario (A) to reconsider the authors' statement: "mating success may not only indicate mate limitation but may also arise from stochasticity and/or individual differences in optimal mating rates (however, if true, we would not expect to observe a non-zero Bateman gradient) [emphasis added]." If we add stochastic mating rates to my scenario (A), then the existing causal arrows can completely produce a non-zero Bateman gradient while the mating rates vary between females. It's just that the variation in reproductive success isn't explained by the additional matings that some females got; it's reflective of them happening to be the larger females who can produce more eggs.

(As an aside, in many phrases in the MS, I would actually welcome a neutral term 'number of matings' rather than 'mating success', as the latter evokes a feeling of succeeding being difficult, i.e. it is an invitation to assume that mate-limitation exists in the system.)

Response:

We fully agree that it is difficult to reconcile the discussed evidence of male harassment and its associated costs on females with the evidence for benefits of mating and the presence of female-female competition at more female-biased sex ratios. However, because we are not experts in guppy reproductive biology, we prefer to avoid drawing strong conclusions by picking just a few studies from the enormous body of work that has been done on guppies. Evaluating the scope for sexual selection to operate in female guppies based on the

literature is presumably a nontrivial endeavor, which probably provides enough material for a stand-alone review article based on a more systematic literature survey, and this is clearly beyond the scope of our contribution. Therefore, we refrain from discussing this species as a showcase example in the manuscript. Nevertheless, this is not to say that we don't find it stimulating and interesting to think about guppies. We do! And we truly appreciate the very detailed pondering, and are very grateful for sharing your thoughts.
As a response to the path diagrams linking body size, mating success and reproductive success, we believe that the most likely scenario for guppies is actually scenario (A) with all arrows being solid – also the one from mating success to reproductive success. As mentioned in our previous response letter, there is (in our view) solid evidence for a causal relationship between mating success and reproductive success. Specifically, various studies experimentally manipulated the number of mating partners and/or the number of matings in guppies and found that multiple mating increased female reproductive success (Evans & Magurran 2000; Ojanguren, Evans & Magurran 2005; Barbosa *et al.* 2012). Combined with the previously mentioned evidence that males have a preference to copulate with larger females which implies a causal link between body size and mating success, we believe that there is empirical evidence suggesting that body size is a sexually selected trait in female guppies. We don't question the presence of male harassment in guppies and evidence for associated costs for females. But even with respect to the costs, the picture might be more complicated than it seems on the first look, because there is also evidence suggesting that females suffer only little (and statistically non-significant) costs from male harassment in terms of fry production, growth rate and foraging (Jerry & Brown 2017). Perhaps one limitation of many studies testing for an effect of male harassment on female reproductive performance is that they often manipulate the sex ratio and not male harassment itself, which makes it tricky to establish a causal relationship with male harassment. This is also true for the highlighted study by Killen *et al.* (2016) in which the sex ratio was manipulated and the frequency of actual male harassment was not quantified. Hence, the observed physiological differences may result from differences in male harassment but may also stem from other effects of the sex ratio treatment (i.e., less female-female aggression in the more male-biased sex-ratio treatment). Interestingly, there is also evidence that female guppies seem to have a preference to group with a shoal of males rather than with a single male or a shoal of females, which is unexpected if females suffer substantial costs from male harassment that override the benefits of multiple mating (Barbosa & Magurran 2011).
All in all, we agree that "Researchers actively state facts against (B)" but there are also several lines of evidence that actually support this scenario. As mentioned in our previous response letter, we are not expert enough on this system to evaluate all the empirical evidence favoring or disfavoring the presence of sexual selection in female guppies. Our personal conclusion from the taken exercise of digging into the guppy literature (probably picking a non-random sample) is that it is complex – and in our view too complex to argue that evidence for male harassment implies that sexual selection on females cannot occur in this species. Whether, why and when females compete for access to mates despite the well-documented costs associated with male harassment are indeed very interesting questions but there might not be an answer to all of them yet. Importantly, we believe that provoking discussions like this one, might be seen as one of the merits of our study. It identifies taxa in which multiple mating in females is positively associated with reproductive success, which then might spur further empirical research trying to understand the underlying causal links and their implications for sexual selection in females.

Specific comments:

--

R1.2: Abstract: No caveats are mentioned here at all (could insert sad emoji here, knowing how many people read the abstract only)

Response: We find it very difficult to cover the caveats associated with the Bateman gradient in an abstract but in the revised version we state explicitly that our evidence is based on a correlation between reproductive success and mating success (L 23-26).

--

Line 173 I would actually say that comparisons across contexts are the most dangerous ones of them all… I think I agree with this sentiment if an "all else being equal" meaning is meant, but across contexts (e.g. sexes), much is typically not equal.

Response: Comparisons across contexts surely require caution but the Bateman gradient together with other standardized metrics of sexual selection (I, Is, smax) have repeatedly been argued to provide means to compare sexual selection across sexes, populations, environments and species (Parker & Birkhead 2013; Mobley 2014; Henshaw & Jones 2019) - something that can hardly be achieved by using a more trait-based approach. We believe that the issue of "all else being equal" is a general issue in comparative studies – though these often are very informative.

--

183 it informs of this if it is not a result of an 'A' type situation

Response: Agreed. We added "at best" to clarify that this statement requires other conditions to be met as well (L 184).

--

223 Here I agree wholeheartedly! See all of above for why I am sometimes confused: you seem to understand this issue so perfectly here… and prefer to not carry it through in other places.

Response: Thanks. We tried to be more careful when drawing conclusions also by adding more "maybes" and "suggests" throughout our manuscript.

--

267 This, to me, appears to indicate that the detection bias (identified by Gerlach) is a real issue (compare this with the response letter's comparison saying why it might not be). Who knows…

Response: We agree that there is a well-known bias when using genetic mating success (Collet *et al.* 2014; Anthes *et al.* 2017). That is why we run an additional analysis restricted to studies using copulatory mating success – which is not subject to this bias and shows that female Bateman gradients are weaker but still positive.

--

300 Now you definitely claim to have demonstrated “strength of selection for mate acquisition” to be the driver of patterns, the caveats are not remembered. Lines 303-304, ditto. Line 305 onwards, now you admit that the above claims actually may or may not be true. I know people should never take individual sentences out of context, but precisely this will happen when using this sort of flow in one’s paragraphs.

Response: We rephrased those sentences trying to use more careful language.

--

315 This made me think of ‘number of mates’, ‘mating rate’ or similar neutral phrases, vs. ‘mating success’ where the latter phrase makes one automatically think there is a directional prediction and that failures are a real possibility. See my comment far above somewhere, in brackets.

Response: Thank you for this interesting suggestion. In fact, copulatory mating success has not only been measured in terms of the number of mating partners but sometimes also as the number of copulations (we added this information in the revised version L 438-440). This makes it inappropriate to replace the term mating success with mating rate or number of mates. Moreover, the term mating success is used in the vast majority of the literature on Bateman’s principles and interested readers can easily find out what ‘mating success’ means as it is clearly defined in the methods section (L 426-449).

--

329-330 Actually, there’s not just these two options. It could also be that there is no mate limitation (for females), also no stochasticity, but a situation (A) as described above, with some females being larger than others and this leading to deterministically more mates for them. The point is that the small females might also have a totally sufficient number of mates, they’re just less fecund because they’re small (and extra mates won’t make them more fecund because this doesn’t change body sizes after all. Note that there are systems where matings yield a lot of resources, e.g. many insects, and then fecundity or even lifespan may actually causally increases with the number of matings!)

Response: Agreed. Many thanks. We added the possibility that variance in mating success can also arise if mating success is associated with another trait that shows variance in the tested populations (L 321).

--

351 …OK, now you do mention this! But would be good to merge with what’s there in 329-

Response: We added this information also in the previous sections (L 329-332).

--

330? Mostly, I think my verbose comments are really about the ‘separation’ between main claims and ‘issues’, if they were better woven into a single coherent argument I’d be really happy about everything.

Response: We tried to be more careful here and at other places throughout the manuscript.

--
378-381 Here you make strong (though implicit) causality claims (you assume ”we can say B, because A has been somehow excluded”)

Response: We rephrased this sentence, separating what our data show from what they suggest (L 379-381).

--
391 Would it actually be honest to mention the complications about one particular species, for illustration of how thorny this is after all? Given that the review process has made us all scratch our heads a lot about guppies, could some of the arguments presented in this interaction be openly discussed here, to provide an illustrative example?

Response: Many thanks for these suggestions. For reasons outlined in the main response, we believe that reviewing the actual empirical evidence favoring and against sexual selection in female guppies is beyond the scope of our article.
We also considered to add path diagrams illustrating under which conditions the Bateman gradient actually informs about the strength of sexual selection on a phenotypic trait. However, this is already covered in detail in previous own work (Anthes *et al.* 2017) and articles by other authors (Henshaw, Jennions & Kruuk 2018) which we are citing in the text and we don’t see how we can provide original material on this issue.

RESPONSES TO REVIEWER 2:

Thank you very much for taking the time to review the current and past versions of our manuscript, and for your helpful suggestions.

--
R2.1: Line 178: Given the possibility for feedbacks (e.g. mate choice copying by females, selection for fecundity by males), the phrasing "is related to" is preferable to "depends on".

Response: Agreed. We changed this (L 179).

--
R2.2: Line 293: "less biased" would be clearer

Response: Agreed. Changed (L 291).

--
R2.3: Line 328: To avoid misreading, clarify that you mean mate limitation experienced by females (competition for males), as opposed to limitation in females

Response: We rephrased this sentence trying to make clear that we mean mate limitation experienced by females (L 328).

REFERENCES

Anthes, N., Häderer, I.K., Michiels, N.K. & Janicke, T. (2017) Measuring and interpreting sexual selection metrics – evaluation and guidelines. *Methods in Ecology and Evolution,* **8,** 918-931.

Barbosa, M., Connolly, S.R., Hisano, M., Dornelas, M. & Magurran, A.E. (2012) Fitness consequences of female multiple mating: A direct test of indirect benefits. *BMC Evolutionary Biology,* **12,** 11.

Barbosa, M. & Magurran, A.E. (2011) Evidence of female-promoted polyandry in Trinidadian guppies. *Environmental Biology of Fishes,* **90,** 95-102.

Collet, J.M., Dean, R.F., Worley, K., Richardson, D.S. & Pizzari, T. (2014) The measure and significance of Bateman's principles. *Proceedings of the Royal Society B-Biological Sciences,* **281,** 9.

Evans, J.P. & Magurran, A.E. (2000) Multiple benefits of multiple mating in guppies. *Proceedings of the National Academy of Sciences of the United States of America,* **97,** 10074-10076.

Henshaw, J.M., Jennions, M.D. & Kruuk, L.E.B. (2018) How to quantify (the response to) sexual selection on traits. *Evolution,* **72,** 1904-1917.

Henshaw, J.M. & Jones, A.G. (2019) Bateman gradient. *Encyclopedia of animal cognition and behavior* (eds J. Vonk & T. Shackelford), pp. 1-4. Springer Nature.

Jerry, M. & Brown, C. (2017) Fitness costs of sexual harassment - the price of persuasion. *Ethology,* **123,** 242-250.

Killen, S.S., Croft, D.P., Salin, K. & Darden, S.K. (2016) Male sexually coercive behaviour drives increased swimming efficiency in female guppies. *Functional Ecology,* **30,** 576-583.

Mobley, K.B. (2014) Mating systems and the measurement of sexual selection. *Animal Behaviour: How and Why Animals Do the Things They Do* (ed. K. Yasukawa), pp. 99-144. Praeger, Santa Barbara.

Nakagawa, S., Lagisz, M., Jennions, M.D., Koricheva, J., Noble, D.W.A., Parker, T.H., Sanchez-Tojar, A., Yang, Y.F. & O'Dea, R.E. (2022) Methods for testing publication bias in ecological and evolutionary meta-analyses. *Methods in Ecology and Evolution,* **13,** 4-21.

Ojanguren, A.F., Evans, J.P. & Magurran, A.E. (2005) Multiple mating influences offspring size in guppies. *Journal of Fish Biology,* **67,** 1184-1188.

Parker, G.A. & Birkhead, T.R. (2013) Polyandry: the history of a revolution. *Philosophical Transactions of the Royal Society B-Biological Sciences,* **368,** 13.
